# Regulation of protein-coding gene and long noncoding RNA pairs in liver of conventional and germ-free mice following oral PBDE exposure

**DOI:** 10.1371/journal.pone.0201387

**Published:** 2018-08-01

**Authors:** Cindy Yanfei Li, Julia Yue Cui

**Affiliations:** Department of Environmental and Occupational Health Sciences, University of Washington, Seattle, Washington, United States of America; University of Navarra School of Medicine and Center for Applied Medical Research (CIMA), SPAIN

## Abstract

Gut microbiome communicates with the host liver to modify hepatic xenobiotic biotransformation and nutrient homeostasis. Polybrominated diphenyl ethers (PBDEs) are persistent environmental contaminants that are detected in fatty food, household dust, and human breast milk at worrisome levels. Recently, long noncoding RNAs (lncRNAs) have been recognized as novel biomarkers for toxicological responses and may regulate the transcriptional/translational output of protein-coding genes (PCGs). However, very little is known regarding to what extent the interactions between PBDEs and gut microbiome modulate hepatic lncRNAs and PCGs, and what critical signaling pathways are impacted at the transcriptomic scale. In this study, we performed RNA-Seq in livers of nine-week-old male conventional (CV) and germ-free (GF) mice orally exposed to the most prevalent PBDE congeners BDE-47 and BDE-99 (100 μmol/kg once daily for 4-days; vehicle: corn oil, 10 ml/kg), and unveiled key molecular pathways and PCG-lncRNA pairs targeted by PBDE-gut microbiome interactions. Lack of gut microbiome profoundly altered the PBDE-mediated transcriptomic response in liver, with the most prominent effect observed in BDE-99-exposed GF mice. The top pathways up-regulated by PBDEs were related to xenobiotic metabolism, whereas the top pathways down-regulated by PBDEs were in lipid metabolism and protein synthesis in both enterotypes. Genomic annotation of the differentially regulated lncRNAs revealed that majority of these lncRNAs overlapped with introns and 3’-UTRs of PCGs. Lack of gut microbiome profoundly increased the percentage of PBDE-regulated lncRNAs mapped to the 3’-UTRs of PCGs, suggesting the potential involvement of lncRNAs in increasing the translational efficiency of PCGs by preventing miRNA-3’-UTR binding, as a compensatory mechanism following toxic exposure to PBDEs. Pathway analysis of PCGs paired with lncRNAs revealed that in CV mice, BDE-47 regulated nucleic acid and retinol metabolism, as well as circadian rhythm; whereas BDE-99 regulated fatty acid metabolism. In GF mice, BDE-47 differentially regulated 19 lncRNA-PCG pairs that were associated with glutathione conjugation and transcriptional regulation. In contrast, BDE-99 up-regulated the xenobiotic-metabolizing Cyp3a genes, but down-regulated the fatty acid-metabolizing Cyp4 genes. Taken together, the present study reveals common and unique lncRNAs and PCG targets of PBDEs in mouse liver, and is among the first to show that lack of gut microbiome sensitizes the liver to toxic exposure of BDE-99 but not BDE-47. Therefore, lncRNAs may serve as specific biomarkers that differentiate various PBDE congeners as well as environmental chemical-mediated dysbiosis. Coordinate regulation of PCG-lncRNA pairs may serve as a more efficient molecular mechanism to combat against xenobiotic insult, and especially during dysbiosis-induced increase in the internal dose of toxicants.

## Introduction

Polybrominated diphenyl ethers (PBDEs) are flame retardants with widespread application in plastics, rubbers, furniture, and electronic devices. Although the industrial use of PBDEs has been recently banned, due to their lipophilic nature, PBDEs are still persistent and bio-accumulative in the environment. Worrisome levels of PBDEs have been detected in human blood (BDE-47: 10~511 ng/g lipid; BDE-99: 2.46~241 ng/g lipid) and breast milk (mean BDE-47: 27.8 ng/g lipid; BDE-99: 5.7 ng/g lipid) [1], raising safety concerns, including neurodevelopmental disorders [2], thyroid toxicity [3], hepatic oxidative stress [4], and carcinogenesis [5]. BDE-47 and BDE-99, which are the predominant PBDE congeners detected in human fluid and fatty diet [6], have been shown to activate the xenobiotic-sensing nuclear receptors pregnane X receptor (PXR) and constitutive androstane receptor (CAR) in rodent livers and human hepatocytes, leading to up-regulated expression of the major drug-metabolizing enzymes cytochrome P450s (Cyps) and other genes involved in xenobiotic biotransformation [7–9].

Liver is a major organ for xenobiotic biotransformation, and increasing evidence in the literature has demonstrated that gut microbiome is a novel frontier for drug metabolism and it communicates with the host liver to fine-tune the efficacy and toxicity of many xenobiotics. Within the gut-liver axis, liver is connected to the gut via enterohepatic circulation, from which the absorbed nutrients and gut microbial metabolites may interact with distinct host receptors in liver to modify various metabolic pathways through remote-sensing mechanisms [10]. We have recently shown that the lack of gut microbiome alters the basal expression of many Cyps and other drug-processing genes (DPGs) in liver, and there is a novel interaction between oral exposure to PBDEs and gut microbiome in modulating the host xenobiotic biotransformation pathways in mouse liver [11, 12]. Specifically, the presence of gut microbiome is necessary in PBDE-mediated transcriptional regulation of many hepatic DPGs, and GF mice had an increase in the hepatic levels of the major BDE-47 hydroxylated metabolite, which is considered more toxic than the parent compound [13]. However, very little is known to what extent gut microbiome and PBDEs modulate intermediary metabolism, which is another important function in liver. Specifically, the liver serves as a central hub for lipogenesis, gluconeogenesis, cholesterol metabolism, bile acid metabolism and protein synthesis [14]. Disrupted intermediary metabolism in the liver is closely associated with inflammatory, proliferative and apoptotic signaling, leading to complex human diseases such as metabolic syndrome and carcinogenesis [15]. Therefore, as a first step to understand the regulation of various metabolic pathways by PBDE-gut microbiome interactions, we used RNA-Seq as a transcriptomic approach to unveil the targeted PCGs in a high-throughput manner.

Long noncoding RNAs (LncRNAs) are increasingly recognized as novel biomarkers and key regulators of toxicological response [16]. LncRNAs are transcribed from the mammalian genome with >200 nucleotides in length but are generally thought to lack protein-coding capability. Various modes of action for lncRNAs have been documented. lncRNAs may serve as scaffolds to transport transcription factors to their promoter region, resulting in transcription activation or repression; function as a ‘decoy’ or ‘molecular sink’ to bind with chromatin regulatory proteins, thereby inhibiting their function [16, 17]; or act *in cis* to activate the transcription of the neighboring genes, whereas others *in trans* may exhibit a suppressive function [18]. Increasing evidence suggests that lncRNAs play a critical role in the regulation of numerous cellular processes, including stem cell pluripotency, development, cell differentiation and apoptosis [19–23]. Aberrant lncRNA expression has been reported in various human diseases such as cancer [23–25]. LncRNAs have been linked to cellular cholesterol metabolism [26], lipid metabolism and bile acid homeostasis [27]. In addition, lncRNAs have been shown to be novel biomarkers for toxic exposure to classic toxicants such as polycyclic aromatic hydrocarbons [28], benzene [29], cadmium [18], and bisphenol A [30]. However, very little is known about the effect of PBDEs and the gut microbiome on the modulation of lncRNA expression in liver, and what potential PCG targets are influenced by differentially regulated lncRNAs.

Therefore, the goal of the present study is to 1) characterize the functional interactions between the gut microbiome and PBDEs and its subsequent changes on the hepatic transcriptome including PCGs and lncRNAs; and more importantly, 2) unveil critical signaling pathways that are targeted by lncRNA-PCG gene pairs with a primary focus on intermediary metabolism and xenobiotic biotranformation pathways. We hypothesize that PBDEs and gut microbiome interact to coordinately regulate PCGs and neighboring lncRNAs that are implicated not only in xenobiotic metabolism but also in intermediary metabolism pathways such as cholesterol and bile acid signaling. This study is among the first to characterize the regulation of lncRNAs and PCGs simultaneously in response to gut microbiome-PBDE interactions, paving the path for future investigations to identify the mechanistic roles of lncRNAs and gut microbiome in PBDE-mediated toxicities.

## Materials and methods

### Animals and procedures

**Fig 1:**
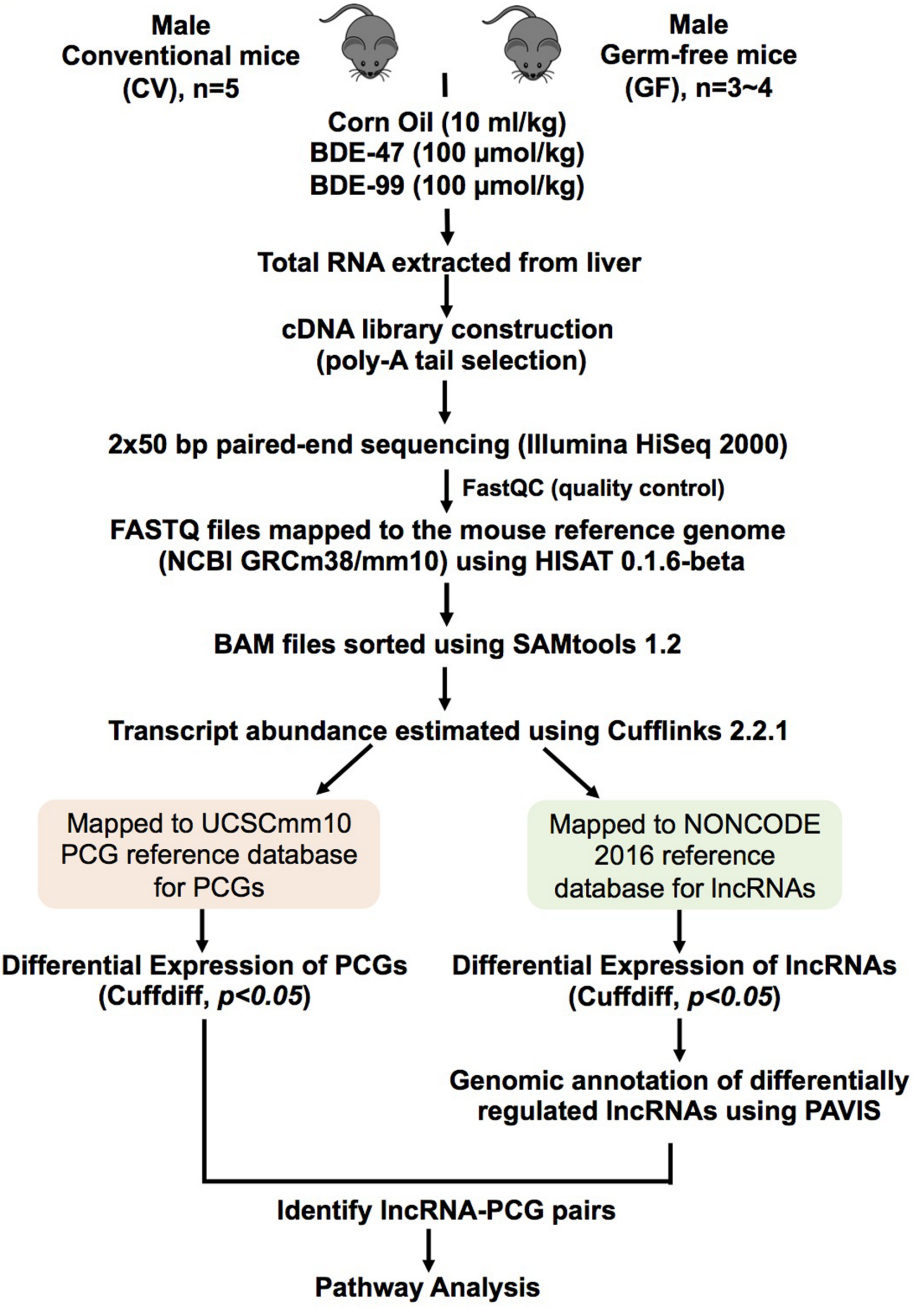
A diagram illustrating the experimental design and workflow for RNA-Seq data analysis. Briefly, 9-week-old male C57BL/6J conventional (CV) and germ-free (GF) mice in C57BL/6 background were exposed to vehicle (corn oil, 10 ml/kg), BDE-47 (100 μmol/kg) or BDE-99 (100 μmol/kg) via oral gavage once daily for 4 consecutive days. Livers were collected 24 hours after the final dose (n = 3~5 per group). Total RNAs were extracted from liver and sent for 50 bp paired-end RNA-Sequencing. RNA-Seq data were quality-checked with FastQC and further analyzed by HISAT, SAMtools, and Cufflinks as described in Materials and Methods. The transcript abundance of protein-coding genes (PCGs) and long non-coding RNAs (lncRNAs) was estimated using the mouse UCSC mm10 PCG and NONCODE 2016 as reference databases, respectively. The differentially expressed PCGs and lncRNAs were analyzed by Cuffdiff between chemical-treated groups and vehicle-treated groups of the same enterotypes of mice (p <0.05). The genomic annotation of differentially regulated lncRNAs in at least one comparison and their closest PCGs were annotated using PAVIS. A lncRNA is considered paired with the neighboring PCG if: 1) lncRNA was transcribed within 5 kb upstream and 1 kb downstream of the neighboring PCG, and 2) both lncRNA and the neighboring PCG were differentially expressed by PBDEs in livers of CV and GF mice (p < 0.05).

As we described previously [13], eight-week-old C57BL/6J wild type conventional (CV) mice were purchased from the Jackson Laboratory (Bar Harbor, ME). The initial breeding colony of GF mice in C57BL/6 background was established with mice purchased from the National Gnotobiotic Rodent Resource Center (University of North Carolina, Chapel Hill). All mice in this study were exposed to the same diet (LabDiet # 5010), bedding (autoclaved Enrich-N’Pure), and water. Mice were housed according to the American Animal Association Laboratory animal care guidelines. As described in Fig 1, at 9-weeks of age, CV and GF mice (n = 3–5 per group) were orally dosed with sterile vehicle (corn oil, 10 ml/kg), BDE-47 (100 μmol/kg) or BDE-99 (100 μmol/kg) once daily for four consecutive days. On the 5^th^ day, which was 24 h after the final dose, mice were euthanized in a CO2 chamber followed by cardiac puncture as described previously [31]. Livers were collected 24 hours after the final dose, immediately frozen in liquid nitrogen, and stored in a -80°C freezer. All studies were approved by the Institutional Animal Care and Use Committee (IACUC) at the University of Washington.

### Total RNA extraction, DNA library construction and RNA-sequencing

RNA extraction, cDNA library construction and RNA-Sequencing were performed as previously described [13]. Briefly, total RNA was extracted from livers of CV and GF mice using RNA-Bee reagent following the manufacturer’s instructions (Tel-Test lnc., Friendswood, TX). RNA concentrations were quantified using a NanoDrop 1000 Spectrophotometer (Thermo Scientific, Waltham, MA). Integrity of total RNA samples was evaluated by Agilent 2100 Bioanalyzer (Agilent Technologies Inc. Santa Clara, CA). Samples with RNA integrity values above 8.0 were sent for RNA-sequencing. The complementary DNA (cDNA) libraries were constructed from total RNA samples using an Illumina TruSeq Stranded RNA kit with Poly-A tail selection (Illumina, San Diego, CA). Sequencing was performed on an Illumina HiSeq2000 sequencer using a 50bp pair-end multiplexing strategy at the University of Washington Genome Sciences Sequencing Facilities.

### RNA-Seq data analysis

The raw and analyzed RNA-Seq data were deposited in Gene Expression Omnibus (GEO) database (accession number: GSE101650), and the FASTQ files were analyzed in the present study for lncRNA and PCG expression. Sample sizes are as follows: CV corn oil: n = 3; CV BDE-47: n = 4; CV BDE-99: n = 2; GF corn oil: n = 3; GF BDE-47: n = 3; GFBDE-99: n = 3. As described in Fig 1, the FASTQ files containing pair-end sequence reads were mapped to the mouse reference genome (GRCm38/mm10) using HISAT (Hierarchical Indexing for Spliced Alignment of Transcripts) (version 0.1.6 beta) [32]. The output SAM (sequencing alignment/map) files were converted to BAM (binary alignment/map) files and sorted using SAMtools (version 1.2) [33]. For the analysis of PCGs and lncRNAs, the transcript abundance was estimated by Cufflinks (version 2.2.1) using the UCSC mm10 PCG and NONCODE 2016 lncRNA reference databases in gene transfer format (gtf), respectively. The mRNA abundance was expressed as fragments per kilobase of transcript per million mapped reads (FPKM). PCGs with average FPKM value above 1 in at least one group were defined as significantly expressed in liver. Differential expression analysis was performed using Cuffdiff with *p* < 0.05 between chemical-exposed groups and vehicle-exposed group of the same enterotypes of mice. Data were expressed as mean FPKM ± S.E. Asterisks (*) represent significant differences between corn oil- and PBDE-treated groups of the same enterotypes of mice. The Venn diagrams of the differentially expressed PCGs and lncRNAs were generated based on their standardized mean FPKM values, using the JMP Genome Software (SAS Institute, Cary, NC). The protocol for the animal experiment as well as the bioinformatics pipeline has been published on protocols.io with the following DOI: doi.org/10.17504/protocols.io.n2tdgen.

### Genomic annotation of lncRNAs and lncRNA-PCG pair identification

The web-based tool peak annotation and visualization (PAVIS, https://manticore.niehs.nih.gov/pavis2/) [34] was used to annotate and visualize the genomic location of lncRNAs relative to the closest PCGs, including up to 5 kb upstream of transcription start site (TSS), intronic, exonic, 5’-untranslated region (UTR), 3’-UTR, or up to 1 kb downstream of transcriptional termination site (TTS). This threshold was set based on the recommended settings of PAVIS. Intergenic regions are defined as lncRNAs located above 5 kb upstream of TSS of PCGs, or over 1kb downstream of PCGs, and do not overlap with any other PCGs. The rationale of utilization this threshold is that as we reviewed recently [35], lncRNAs can function together with transcription factors forming transcriptional scaffold to increase gene transcription. Although we have previously showed that the genomic interactions could occur outside this range using wet-lab approach [36], we chose this stringent threshold to reduce the false-positive observations using computational approach.

A lncRNA-PCG pair meets the following criteria: 1) the lncRNA overlaps with or is within 5 kb upstream of TSS or 1 kb downstream of TTS of its closest PCG, and 2) both the PCG and the neighboring lncRNA are differentially regulated by PBDE exposure compared to vehicle-treated group of the same enterotypes of mice (*p* < 0.05). The gene structure and relative genomic location of the lncRNA-PCG pairs are visualized using Integrated Genome Viewer (Broad Institute, Cambridge, MA).

### Pathway analysis of differentially regulated PCGs and lncRNAs

The differentially regulated PCGs by 1) BDE-47 in CV mice, 2) BDE-99 in CV mice, 3) BDE-47 in GF mice or 4) BDE-99 in GF mice were each analyzed using Ingenuity Pathway Analysis (IPA, QIAGEN, Valencia, CA) to determine significantly altered gene networks. The PCGs that were uniquely regulated by each of the above four cases were further analyzed by IPA to determine the specific effect of BDE-47 and BDE-99 in CV and GF conditions. LncRNAs that were differentially regulated in each of the above four cases were separated into four datasets, and their paired PCGs in each dataset were submitted for STRING (Search Tool for the Retrieval of Interacting Genes/Proteins) analysis (https://string-db.org/).

## Results and discussion

The present study utilized RNA-Seq and *in vivo* CV and GF mouse models to determine the effect of functional interactions between the gut microbiome and PBDEs on the hepatic transcriptome of PCGs and lncRNAs. RNA-Seq generated approximately 39 to 127 million reads per sample, with 63% to 94% of the reads were mapping uniquely to the mouse reference genome (NCBI GRCm38/mm10), which were equivalent to 35 million to 115 million uniquely mapped reads (S1 Table).

### Regulation of PCGs by PBDEs and gut microbiome

**Fig 2:**
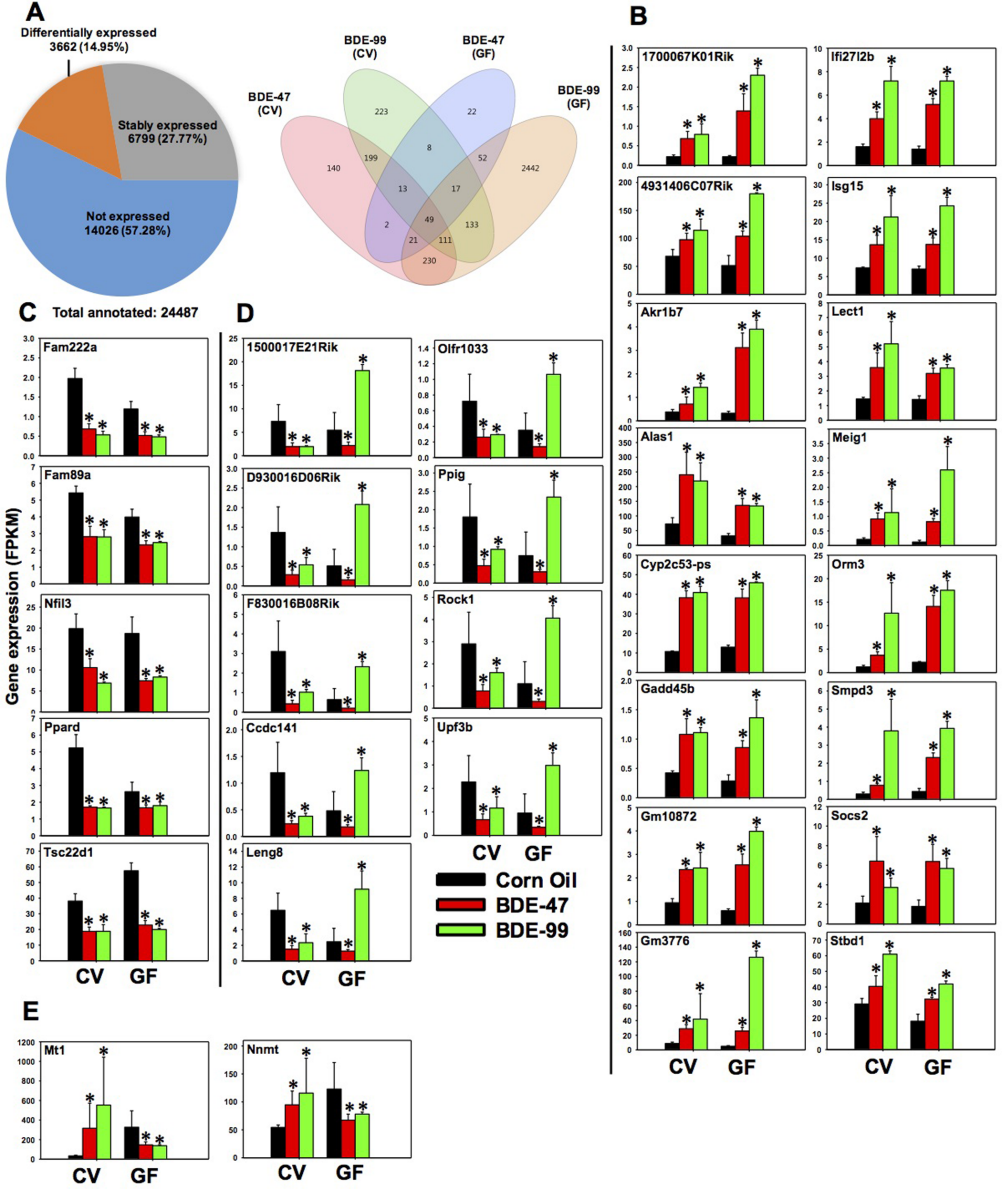
Regulation of protein-coding genes (PCGs) in livers of CV and GF mice exposed to corn oil, BDE-47 (100 μmol/kg), or BDE-99 (100 μmol/kg). **A.** Left: a pie chart showing PCGs that were not expressed in any treatment groups (blue), stably expressed in all groups (grey), and differentially expressed by PBDE exposure in at least one of the four comparisons (orange). These four comparisons are: CV_CO (corn oil) vs CV_BDE-47; CV_CO vs CV_BDE-99; GF_CO vs GF_BDE-47; and GF_CO vs GF_BDE-99, FDR adjusted p-value <0.05). Right: a Venn diagram showing the differentially expressed PCGs that were commonly or uniquely regulated by PBDEs among the four comparisons as described in Fig 2A. Venn diagram was generated using JMP Genomics. **B-E:** PCGs that were differentially regulated by both PBDEs in livers of CV and GF mice. **B.** PCGs that were commonly up-regulated by BDE-47 and BDE-99 in livers of both CV and GF mice. **C.** PCGs that were commonly down-regulated by BDE-47 and BDE-99 in livers of both CV and GF mice. **D.** PCGs that were consistently down-regulated by BDE-47 in livers of CV and GF mice, but were oppositely regulated in CV (down-regulation) and GF (up-regulation) mice by BDE-99. **E.** PCGs that were oppositely regulated in CV (up-regulation) and GF (down-regulation) mice by both BDE-47 and BDE-99. Asterisks (*) indicate statistically significant differences as compared to vehicle-treated groups of the same enterotypes of mice (Cuffdiff, *p* < 0.05).

Among the 24,487 annotated PCGs in the mouse reference genome (Fig 2A), 14,026 genes were not expressed in livers of any groups (threshold: average FPKM < 1 in all treatment groups). Among the expressed genes (FPKM> 1 in at least one treatment group), 6,799 genes were stably expressed across all treatment groups, whereas a total of 3,662 genes were differentially expressed by PBDEs in CV and GF mice (criteria: *p* < 0.05 in at least one of the 4 comparisons between corn oil (CO) and PBDE-exposed groups of the same enterotype, namely 1) corn oil exposed CV mice (CV_CO) vs. CV_BDE-47, 2) CV_CO vs. CV_BDE-99, 3) corn oil exposed GF mice (GF_CO) vs. GF_BDE-47, and 4) GF_CO vs. GF_BDE-99).

To unveil common and unique PCG targets by PBDEs and gut microbiome interactions, a Venn diagram of the 3,662 differentially expressed PCGs was generated, which revealed a clear separation in differences in both PBDE congeners (BDE-47 or BDE-99) and enterotypes (presence or absence of gut microbiome) (Fig 2A). There were 49 PCGs that were consistently differentially regulated in all 4 comparisons. Uniquely regulated DPGs in each comparison were also observed, and especially the number of PCGs uniquely regulated by BDE-99 in GF conditions (2442) was much higher than that in CV conditions (223), suggesting that the lack of gut microbiome sensitized the hepatic protein-coding transcriptome to BDE-99 mediated insult. Conversely, 140 PCGs and 22 PCGs were uniquely regulated by BDE-47 in CV and GF conditions, respectively. Therefore, the two PBDE congeners lead to different transcriptomic outcomes during PBDE-gut microbiome interactions.

To determine the top differentially regulated PCGs and the associated signaling networks, Ingenuity Pathway Analysis (IPA) was performed for the differentially regulated PCGs (*p* < 0.05) among the 4 comparisons (CV_CO vs. CV_BDE-47, CV_CO vs. CV_BDE-99, GF_CO vs. GF_BDE-47, and GF_CO vs. GF_BDE-99).

#### BDE-47 and livers of CV mice

As shown in Table 1, the top 10 most up-regulated PCGs in livers of CV mice following BDE-47 exposure include the phase-I drug-metabolizing enzymes cytochrome P450 (Cyp) family, namely Cyp2b10, Cyp2b13, and Cyp2c55; metallothionein (Mt) 1 and Mt2, which are involved in zinc homeostasis and heavy metal detoxification [37]; D site albumin promoter binding protein (Dbp) and circadian associated repressor of transcription (Ciart, also known as Gm129), which modulate important clock genes involved in circadian rhythm [38, 39]; regulator of G-protein signaling 16 (Rgs16), which inhibits signaling cascades related to fatty acid oxidation in hepatocytes [40]; serum amyloid A2 (Saa2), which is an apolipoprotein and is highly expressed in liver in response to inflammation [41]; and lipocalin 2 (Lcn2), which is an iron-trafficking protein that maintains iron homeostasis [42]. Hepatocyte-derived Lcn2 has also been reported to protect against bacterial infection and promote liver regeneration [43]. In livers of CV mice, the top 10 most down-regulated PCGs following BDE-47 exposure include growth arrest and DNA damage 45g (Gadd45g) and proline-rich acidic protein 1 (Prap1), which are involved in regulation of development, cell differentiation and survival [44, 45]; trefoil factor 2 (Tff2), which mediates gastric cytoprotection and repair [46]; regenerating islet-derived 3 beta (Reg3b) and deleted in malignant brain tumors 1 (Dmbt1), which maintain intestinal homeostasis and prevent bacterial infection [47, 48], as well as neuronal PAS domain protein 2 (Npas2), which functions as a transcription activator involved in circadian rhythms [49] (Table 1).

**Table 1:** Top PCGs that were differentially regulated by PBDEs in livers of CV and GF mice compared to the vehicle-treated control group of the same enterotype of mice.

Treatment	Up-regulated		Down-regulated	
	Gene	Fold-increase	Gene	% decrease
CV_CO vs CV_BDE-47	Mt2	37.29	Gadd45g	82%
	Cyp2b10	18.84	Tff2	82%
	Dbp	14.68	Reg3b	83%
	Cyp2b13	14.38	Prap1	83%
	Cyp2c55	13.45	F830016B08Rik	86%
	Rgs16	11.67	Firre	87%
	Saa2	11.17	Neat1	88%
	Lcn2	10.65	Spink3	90%
	Ciart	10.16	Npas2	94%
	Mt1	9.34	Dmbt1	95%
CV_CO vs CV_BDE-99	Cel	533.16	Cdkn1a	84%
	Saa2	389.22	Loxl4	84%
	Ctrb1	334.83	Hcn3	85%
	Rnase1	322.47	Chka	86%
	Cpb1	320.53	Elovl3	86%
	Prss2	284.89	Clic3	87%
	Try5	274.07	Serpina4-ps1	89%
	Prss3	268.69	Gadd45g	92%
	Pnlip	266.54	Arntl	94%
	Lcn2	253.64	Npas2	99%
GFCO vs GF_BDE-47	Sult2a2	25.39	Serpina9	64%
	Sult2a1	24.13	Atrx	64%
	Cyp2b10	22.41	Phip	67%
	Cyp2c55	15.88	F830016B08Rik	68%
	Cyp2b13	12.71	D930016D06Rik	69%
	Akr1b7	9.66	Igip	69%
	A1bg	8.50	Rock1	72%
	Fmo3	8.38	Malat1	75%
	Cyp3a16	7.87	Bcl6	79%
	Pnliprp1	7.66	Snora7a	82%
GFCO vs GF_BDE-99	Cyp2b10	53.56	Tff3	72%
	Cyp2c55	52.83	H2-Ab1	73%
	Meig1	21.79	Flywch2	74%
	Gsta1	19.15	Hbb-bt	75%
	Gstm3	18.41	Serpina3c	76%
	Sult1e1	15.48	Cyp46a1	77%
	Vtcn1	15.42	Rad51b	79%
	Pnliprp1	15.39	Serpina12	82%
	Snhg11	12.46	Acot1	83%
	Akr1b7	12.08	Sult2a1	100%

In livers of CV mice, the top 15 networks of PCGs that were differentially regulated by BDE-47 exposure are shown in S2 Table. Interestingly, consistent with our hypothesis, BDE-47 exposure not only altered many known xenobiotic biotransformation pathways in livers of CV mice, but also many intermediary metabolism pathways, such as lipid metabolism, carbohydrate metabolism, as well as host defense pathways such as immune functions (S2 Table). An example network (lipid and drug metabolism, Network #14 as shown in red in S2 Table) is visualized in S1 Fig. BDE-47 markedly up-regulated Cyp2b family members that are involved in xenobiotic metabolism. BDE-47 also up-regulated Cyp7a1 and Cyp7b1, which are responsible for bile acid synthesis, which is a major mechanism for cholesterol elimination [50]; conversely, BDE-47 down-regulated Cyp4a14, which is the prototypical target gene of lipid sensor peroxisome proliferator-activated receptor alpha (PPAR*α*) and is involved in fatty acid oxidation [51], suggesting that BDE-47 may activate the xenobiotic oxidation and bile acid synthesis but suppress the hydroxylation of fatty acids and lipid metabolism signaling.

#### BDE-99 and livers of CV mice

As shown in Table 1, the top 10 most up-regulated genes in livers of CV mice following BDE-99 exposure were also important for intermediary metabolism. These include carboxyl ester lipase (Cel) and pancreatic lipase (Pnlip), which catalyze fat digestion/absorption and glycerolipid metabolism [52, 53]; BDE-99 also up-regulated genes such as chymotrypsinogen B1 (Ctrb1), carboxypeptidase B1 (Cpb1), protease serine 2 (Prss2), Prss3, and trypsin 5 (Try5), which are involved in protein digestion and absorption [54]; ribonuclease (Rnase1), which catalyzes the cleavage of RNA [55]; and Saa2 and Lcn2, which suppress bacterial infection [41, 43]. Interestingly, the fold increases of the top 10 PCG transcripts by BDE-99 exposure were in general much greater than those of the top 10 PCG transcripts by BDE-47 exposure, suggesting that at equal molar doses, BDE-99 is more potent than BDE-47 in up-regulating the hepatic PCGs expression (Table 1). The top 10 most down-regulated PCGs in the livers of CV mice following BDE-99 exposure include cyclin-dependent kinase inhibitor 1A (Cdkn1a) and Gadd45g, which are involved in p53 signaling in response to DNA damage [56]; lysyl oxidase-like 4 (Loxl4), which modulates the formation of extracellular matrix [57]; hyperpolarization-activated cyclic nucleotide-gated channel (Hcn3), which is a potassium channel and controls cellular excitability [58]. BDE-99 also down-regulated certain PCGs involved in circadian rhythm, such as the transcriptional activator Arntl (Aryl hydrocarbon receptor nuclear translocator-like), the transcriptional repressor Npas2, as well as Chka (choline kinase alpha), which is regulated by the clock transcription factor REV-ERB [59]. In addition, elongation of very long chain fatty acids (Elovl3), as well as chloride intracellular channel 3 (Clic3), which forms chloride ion channels and controls cellular growth, were down-regulated by BDE-99 exposure in livers of CV mice (Table 1).

Similar to BDE-47, BDE-99 exposure also altered both xenobiotic and intermediary metabolism (such as lipid and carbohydrate) pathways, as well as immune functions (S2 Table). An example network (#13) of lipid metabolism, small molecule biochemistry, vitamin and mineral metabolism is shown in S2 Fig. In this network, a variety of P450s including Cyp2a, Cyp2b, Cyp2c, Cyp2g1, Cyp3a and the cytochrome P450 oxidoreductase (Por), were all moderately up-regulated by BDE-99. Similar to BDE-47, BDE-99 also up-regulated Cyp7b1, which is involved in the alternative bile acid synthesis pathway, but down-regulated Cyp8b1, which is involved in the classic bile acid synthesis pathway. BDE-99 also down-regulated many genes involve in lipid metabolism and fatty acid degradation, including a consistent down-regulation of PPARα-targeted Cyp4 family members (Cyp4a11, Cyp4a14, Cyp4f14 [mouse homolog of human CYP4F12]); Cyp46a1, which is the rate-limiting enzyme for cholesterol degradation [60]; 3-hydroxymethylglutaryl-CoA synthase (Hmgcs1), which forms the intermediate HMG-CoA for cholesterol synthesis and ketogenesis [61]; and acyl-CoA synthetase (Acsl), which activates long-chain fatty acids for both synthesis of cellular lipids and degradation via beta-oxidation [62]. In addition, BDE-99 down-regulated Cyp26a1, which is involved in the metabolism of retinoic acid [63].

In summary, in CV conditions, both BDE-47 and BDE-99 regulate many PCGs involved in intermediary metabolism in liver.

#### BDE-47 and livers of GF mice

As shown in Table 1, in livers of GF mice, the top 10 most up-regulated genes following BDE-47 exposure include many phase I and phase II drug metabolizing enzymes, such as Sult2a2, Sult2a1, Cyp2b10, Cyp2c55, Cyp2b13, Akr1b7, Fmo3, and Cyp3a16; BDE-47 also up-regulated alpha-1-B glycoprotein (A1bg), and pancreatic lipase protein 1 (Pnliprp1), which has been associated with dietary lipid absorption and fatty liver development [64]. Compared to CV conditions exposed to the same chemical, as expected, there were more PCGs in the top 10 gene list that are involved in xenobiotic detoxification pathways.

The top 10 most down-regulated PCGs in the livers of GF mice following BDE-47 exposure include serine peptidase inhibitor member 9 (Serpina9); alpha thalassemia/mental retardation syndrome X-linked homolog (Atrx), which is involved in transcriptional regulation and chromatin remodeling [65]; pleckstrin homology domain interacting protein (Phip), which modulates insulin signaling [66]; IgA inducing protein (Igip), which induces the production of IgA in maintaining gut homeostasis [67]; Rho-associated protein kinase 1 (Rock1), which regulates actin cytoskeleton organization, cell motility, proliferation and apoptosis [68]; B cell leukemia/lymphoma 6 (Bcl6), which functions as a transcriptional repressor in modulating interleukin-4 signaling [69]; and small nucleolar RNA 7A (Snora7a), which is involved in cell proliferation especially in tumor cells [70].

The top 12 networks of PCGs that were differentially regulated in the livers of GF mice by BDE-47 exposure are shown in S2 Table. Consistent with the top 10 gene list described in Table 1, the pathway “drug metabolism, glutathione depletion in liver, and lipid metabolism” ranked the top most differentially regulated pathway within the entire liver transcriptome in livers of GF mice exposed to BDE-47. This is likely due to compensatory up-regulation of the hepatic defense mechanism to combat against increasing amount of toxic oxidative metabolites of BDE-47, in the absence of gut microbiome which performs the majority of reduction reactions. This network is visualized in S3 Fig. BDE-47 markedly up-regulated Cyp2b10 (mouse homolog of human CYP2B6) and Cyp3a11 (mouse homolog of human CYP3A5) that are involved in xenobiotic metabolism. BDE-47 also up-regulated Cyp2a, Cyp2c54, Cyp2c55 (mouse homolog of human CYP2C18), and many other Cyp2c family members. The glutathione *S*-transferases (Gst), including Gsta4, Gsta5, Gstm3-5, and Gstt3 that are important for the detoxification and reduction of reactive oxygen species and electrophiles, were also up-regulated by BDE-47 in GF mice.

#### BDE-99 and livers of GF mice

The top 10 most up-regulated PCGs in livers of GF mice following BDE-99 exposure are involved in xenobiotic metabolism, immune response, as well as RNA and cell proliferation pathways. Specifically, these genes include drug-metabolizing enzymes Cyp2b10, Cyp2c55, Gsta1, Gstm3, Sult1e1, and Akr1b7; meiosis expressed gene 1 (Meig1); V-set domain containing T cell activation inhibitor 1 (Vtcn1, also known as B7-H4), which suppresses T cell function via inhibiting cell proliferation and cytokine secretion [71], Pnliprp1, as well as small nucleolar RNA host gene 11 (Snhg11). Interestingly, the fold increases of the top 10 PCG transcripts by BDE-99 exposure were in general much greater than those of the top 10 PCG transcripts by BDE-47 exposure, again suggesting that at equal molar doses, BDE-99 is more potent than BDE-47 in up-regulating the hepatic PCGs expression (Table 1). The top 10 most down-regulated genes in the livers of GF mice following BDE-99 exposure included trefoil factor 3 (Tff3) [72]; histocompatibility 2, class II antigen A, beta 1 (H2-Ab1); FLYWCH family member 2 (Flywch2); hemoglobin, beta adult t chain (Hbb-bt), which transports oxygen to peripheral tissues; serine peptidase inhibitor member 3C (Serpina3c); Serpina12; Cyp46a1, which is the rate-limiting enzyme for cholesterol degradation [60]; RAD51 homolog b (Rad51b), which is involved in DNA repair [73]; and acyl-CoA thioesterase 1 (Acot1), which catalyzes the hydrolysis of acyl-CoAs to free fatty acid and Coenzyme A. Knockdown of Acot1 in mice resulted in enhanced hepatic oxidative stress and inflammation [74]. Additionally, Sult2a1, which catalyzes sulfonation of hydroxysteroids and xenobiotics, was completely abolished in livers of GF mice following BDE-99 exposure.

In livers of GF mice, the top 15 networks of PCGs that were differentially regulated by BDE-99 exposure are shown in S2 Table, which include pathways related to cancer, developmental disorder, RNA post-transcriptional modification, metabolic disease, carbohydrate metabolism, gene expression, protein synthesis. An example network (gene expression, protein synthesis, lipid metabolism, network #8 as shown in red in S2 Table) is visualized in S4 Fig. BDE-99 markedly down-regulated a series of mitochondrial ribosomal proteins (Mrpl), which are essential for oxidative phosphorylation to produce ATP [75]. This massive down-regulation of Mrpl by BDE-99 may lead to oxidative phosphorylation disorder and various mitochondrial diseases [76].

In summary, both PBDE exposure and gut microbiome regulated the hepatic transcriptome in mice, and lack of gut microbiome appeared to sensitize mouse liver to BDE-99-mediated effects on the transcriptome of PCGs.

### PCGs that were commonly or uniquely regulated by PBDEs in CV and GF mice

#### Commonly regulated PCGs

Regarding the 49 consistently differentially regulated genes (Fig 2A Venn Diagram), 32 of them are shown in Fig 2B; whereas the remaining 17 genes (including Cyp2a, Cyp2b, Cyp2c, Ugt2b, Sults and Gsts) have been described previously in our recent publication [31]. All of these genes were up-regulated by BDE-47 and BDE-99 in livers of CV and GF mice. KEGG pathway analysis (performed using STRING Analysis) of these 32 commonly regulated genes indicates that they are enriched in metabolic pathway, chemical carcinogenesis, retinol metabolism, steroid hormone biosynthesis, and arachidonic acid metabolism (S5 Fig). As shown in Fig 2B–2E, these genes were categorized into four distinct expression patterns. Pattern 1 includes 16 genes (Fig 2B), which were up-regulated by both PBDEs in CV and GF mice, including RIKEN-derived genes (1700067K01Rik and 4931406C07Rik); Aldo-keto reductase (Akr1b7) which is a phase-I drug metabolizing enzyme [77]; animolevulinic acid synthase 1 (Alas1), which is the rate-limiting enzyme for heme synthesis in liver [78], and human hepatic ALAS1 is the target gene of the bile acid-activated nuclear receptor farnesoid X receptor (FXR) [79]; Cyp2c53-ps (Cyp pseudogene); Gadd45b; Gm10872; Gm3776; interferon alpha-inducible protein 27 like 2B (Ifi27l2b); interferon-stimulated gene 15 (Isg15); leukocyte cell derived chemotaxin 1 (Lect1); Meig1; orosomucoid 3 (Orm3), which is expressed in hepatocytes under stress condition and is a direct FXR target gene [80]; sphingomyelin phosphodiesterase 3 (Smpd3), which produces ceramide for cell growth, differentiation, and apoptosis [81]; suppressor of cytokine signaling 2 (Socs2), which regulates hepatic homeostasis under high fat diet treated conditions [82]; and starch-binding domain 1 (Stbd1), which participates the metabolism and cellular trafficking of glycogen [83]. Pattern 2 included 5 genes (Fig 2C), which were down-regulated by both PBDEs in CV and GF mice, including family with sequence similarity genes (Fam222a); Fam89a; nuclear factor interleukin 3 (Nfil3), which plays a critical role in the regulation of apoptosis in lymphocytes [84]; Ppard, which suppresses hepatic lipogenesis in obese mice [85]; and transforming growth factor beta-stimulated clone 22 homolog (Tsc22d1), which controls systemic cholesterol metabolism. Overexpression of Tsc22d1 promotes high levels of high-density lipoprotein (HDL) cholesterol in mice [86]. Pattern 3 included 9 genes (Fig 2D), which were consistently down-regulated by BDE-47 in CV and GF mice, but were down-regulated by BDE-99 in CV mice and up-regulated by BDE-99 in GF mice. These genes included RIKEN-derived genes (1500017E21Rik, D930016D06Rik, F830016B08Rik), coiled-coil domain containing 141 (Ccdc141), leukocyte receptor cluster member 8 (Leng8), olfactory receptor 1033 (Olfr1033), peptidylprolyl isomerase G (Ppig), Rock1, and up-frameshift suppressor 3 homolog B (Upf3b). Pattern 4 consisted of 2 genes (Fig 2E), which were up-regulated by both BDE-47 and BDE-99 in CV mice, but were down-regulated in GF mice. These two genes are Mt1, which is involved in zinc homeostasis and heavy metal detoxification [37], and nicotinamide N-methyltransferase (Nnmt), which regulates hepatic nutrient metabolism [87]. The PBDE-mediated up-regulation of Mt1 and Nnmt only occur with the presence of gut microbiome suggests a gut microbiome dependent manner.

#### Uniquely regulated PCGs

Regarding the unique effect of BDE-47 in CV mice, the top 10 most up-regulated PCGs by BDE-47 are shown in Table 2, including Gm7694; major urinary protein family members (Mup12 and Mup8), which regulate glucose and energy metabolism [88], oligoadenylate synthases (Oas1a and Oas1g), which regulate the early phase of viral infection and inhibit viral replication [89]; 2010003K11Rik; calcium and integrin binding protein 3 (Cib3), which is involved in the integrin signaling pathway [90]; phospholipid scramblase 1 (Plscr1), which is required for maturation and differentiation of hematopoietic cells from progenitor cells [91]; membrane-spanning 4-domains subfamily A member 6D (Ms4a6d), which regulates signal transduction [92]; and Cyp4a12b, which is important for fatty acid oxidation [93]. The top 10 most down-regulated genes by BDE-47 exclusively in the livers of CV mice included zinc finger protein (Zfp935); small nucleolar RNA host gene 5 (Snhg5), which is up-regulated in colorectal cancer and causes tumor outgrowth *in vivo* [94]; mitogen-activated protein kinase 8 interacting protein 3 (Mapk8ip3, also known as Jip3), which is involved in JNK signaling pathway and down-regulates oxidative stress and inflammatory response in high fat diet-treated mice [95]; Ras related oncogene family member (Rab26os), which modulates intracellular membrane trafficking [96]; AT-rich interaction domain 4B (Arid4b), which is a subunit of transcriptional repressor complex and involves in cell proliferation, differentiation, apoptosis and cell fate determination [97]; Ly6/PLAUR domain containing 8 (Lypd8), which prevents microbiota invading the colonic epithelia in mice [98]; pre-mRNA processing factor (Prpf39), which correlates with cisplatin cytotoxicity and knockdown of Prpf39 results in a significant increase in cisplatin resistance [99]; and proline rich acidic protein 1 (Prap1), which maintains normal growth homeostasis in epithelial cells [45].

**Table 2:** Top PCGs that were uniquely regulated by BDE-47 in CV condition, by BDE-99 in CV condition, by BDE-47 in GF condition, and by BDE-99 in GF condition.

Treatment	Up-regulated		Down-regulated	
	Gene	Fold-increase	Gene	% decrease
CV_CO vs CV_BDE-47	Gm7694	2.20	Zfp935	62%
	Mup12	2.07	Snhg5	63%
	Oas1g	2.04	Mapk8ip3	65%
	2010003K11Rik	1.96	Rab26os	66%
	Mup8	1.96	Arid4b	69%
	Oas1a	1.84	Lypd8	70%
	Cib3	1.84	Lgals4	75%
	Plscr1	1.81	Rbp2	76%
	Ms4a6d	1.75	Prpf39	78%
	Cyp4a12b	1.74	Prap1	83%
CV_CO vs CV_BDE-99	Cel	533.16	Fpgs	56%
	Ctrb1	334.83	Ndrg1	56%
	Rnase1	322.47	Szt2	56%
	Cpb1	320.53	Id3	58%
	Prss2	284.89	Elovl5	58%
	Try5	274.07	Phospho1	59%
	Prss3	268.69	Nedd4l	60%
	Pnlip	266.55	Mypop	60%
	2210010C04Rik	240.82	Ngfr	62%
	Cpa1	227.64	Tubb2b	83%
GFCO vs GF_BDE-47	Sult2a2	25.39	Got1	31%
	A1bg	8.50	Sesn2	38%
	Ptgds	5.64	Epha2	41%
	Gmds	3.41	Dct	43%
	Mup4	2.65	Snora7a	82%
	Acot3	2.11		
	Fam213b	2.08		
	Eif4ebp3	2.04		
	Cyp39a1	1.96		
	Mup19	1.88		
GFCO vs GF_BDE-99	Sult1e1	15.48	Ccl5	63%
	Gm11974	8.62	Camk2b	64%
	Mmd2	5.86	Map1lc3a	64%
	Gm14403	5.70	Caln1	67%
	Hsp90aa1	4.52	Ces4a	69%
	Zfp445	4.40	Wfdc2	70%
	Samd9l	4.37	Guca1a	72%
	Aox1	4.24	H2-Eb1	72%
	Robo1	4.01	Flywch2	74%
	Slc10a2	3.90	Serpina3c	76%

Ingenuity Pathway Analysis (IPA) was performed for PCGs that were uniquely regulated following BDE-47 exposure in CV mice, and is shown in S6 Fig, which include pathways related to cellular development, cancer, cell death and survival, carbohydrate metabolism, lipid metabolism, and inflammatory response. An example network (cellular development, cancer, cell death and survival, network #1) is visualized in S6 Fig. BDE-47 up-regulated Iron-binding nuclear protein (Pir), Gstp1, and heme oxygenase 1 (Hmox1), which protect cells from oxidative stress [100, 101]. BDE-47 also up-regulated mothers against decapentaplegic homolog 9 (Smad9; also known as Smad8), which modulates transforming growth factor beta (TGF-β) cellular signaling during proliferation, differentiation and development [102]; guanylate binding protein 2 (Gbp2), which alters cell proliferation and inhibits cell spreading [103]; and hairy and enhancer of split-1 (Hes1), which encodes a transcription repressor that influences cell proliferation and differentiation during embryogenesis [104]. Conversely, BDE-47 down-regulated lectin, galactoside-binding soluble 4 (Lgals4), which inhibits cell proliferation as a tumor suppressor [105]; polycystin 2 (Pkd2), which transports calcium ions into the cell and regulates cell growth and division, cell movement, and cell-cell interaction [106]; and retinol binding protein 2 (Rbp2), which participates in the uptake and intracellular metabolism of vitamin A for cell differentiation, proliferation, and apoptosis [107].

Regarding the unique effect of BDE-99 in CV mice, the top 10 most up-regulated genes by BDE-99 are shown in Table 2, including Cel, which is important for cholesterol and lipid-soluble vitamin ester hydrolysis and absorption [52]; Ctrb1, Rnase1, Cpb1, Prss2, Try5, and Prss3. BDE-99 also up-regulated Pnlip, which hydrolyzes triglycerides in the small intestine and digests dietary fats [108], 2210010C04Rik, and carboxypeptidase A1 (Cpa1). Conversely, the top 10 most down-regulated genes by BDE-99 in CV mice included folylpolyglutamyl synthetase (Fpgs), which maintains folate homeostasis for cell proliferation [109]; N-myc downstream regulated gene 1 (Ndrg1), which responds to cellular stress signals [110]; seizure threshold 2 (Szt2), which controls cell growth [111]; inhibitor of DNA biding 3 (ld3), which inhibits transcription [112]; ELOVL fatty acid elongase 5 (Elovl5); phosphoethanolamine/phosphocholine phosphatase (Phospho1), which is important for skeletal development; Myb related transcription factor (Mypop), which regulates hematopoiesis and tumorigenesis [113]; nerve growth factor receptor (Ngfr); and tubulin beta 2B class IIb (Tubb2b), which encodes tubulin that is major component of microtubules [114]. Ingenuity pathway analysis (IPA) was performed for the genes that were uniquely regulated following BDE-99 exposure in CV mice, and is shown in S7 Fig, which includes carbohydrate metabolism, lipid metabolism, small molecule biochemistry, post-translational modification, protein degradation, protein synthesis, cancer, and cellular assembly and organization. Examples of the top two networks are visualized in S7 Fig. For example, within the network of carbohydrate metabolism and lipid metabolism, BDE-99 down-regulated regulatory factor X4 (Rfx4); MYCL proto-oncogene (Mycl); persulfide dioxygenase (Ethe1), which modulates sulfide detoxification and regulates mitochondrial catabolism of fatty acids [115]; Forkhead Box protein A2 (Foxa2), which regulates lipid metabolism and ketogenesis in the liver during fasting and in diabetes [116]; mediator complex subunit 24 (Med24), which is involved in adipogenesis [117]; and Neural precursor cell expressed, developmentally down-regulated gene 4-like (Nedd4l), which is associated with diabetes and obesity [118]. Conversely, BDE-99 markedly up-regulated the digestive enzyme Rnase1 and pnlip, which hydrolyze triglycerides in the small intestine and digests dietary fats [108]. Within the network of post-translational modification, BDE-99 also up-regulated a series of genes involved in protein degradation such as serine protease, trypsinogen, and chymotrypsin, but down-regulated many collagen-related protein synthesis, such as collagen type V alpha 1 chain (Col5a1), Col3a1, ERBB receptor feedback inhibitor 1 (Errfl1),serpin family H member 1 (Serpinh1), and phospholipase C beta 3 (Plcb3).

Regarding the unique effect of BDE-47 in GF mice, the top 10 most up-regulated genes are shown in Table 2, including Sult2a2, which is a bile salt sulfotransferase that catalyzes sulfonation of hydroxysteroids [119]; alpha-1B glycoprotein (A1bg); prostaglandin D2 synthase (Ptgds); GDP-mannose 4,6-dehydratase (Gmds); Mup4; Acyl-CoA thioesterase 3 (Acot3), which catalyzes the hydrolysis of acyl-CoAs to free fatty acid [120]; Fam213b; Eukaryotic translation initiation factor 4E binding protein 3 (Eif4ebp3); Cyp39a1, which catalyzes the hydroxylation of cholesterol in the alternative bile acid synthesis pathway [121]; and Mup19. Conversely, the top most down-regulated genes by BDE-47 included glutamic-oxaloacetic transaminase 1 (Got1), which is important for amino acid metabolism and the urea and tricarboxylic acid cycles [122]; Sestrin 2 (Sesn2) and dopachrome tautomerase (Dct), both of which protect cells from oxidative stress [123, 124]; ephrin type-A receptor A2 (Epha2), which mediates neural development [125]; and small nucleolar RNA 7A (Snora7a). Snora7a has been reported to promote cell proliferation and suppress differentiation in mesenchymal stem cell [126]. These results suggested that without the presence of gut microbiome, not only steroid and bile acid signaling pathways, but the cellular signaling were distinctly regulated by BDE-47 in GF mice.

Regarding the unique effect of BDE-99 in GF mice, the top 10 most up-regulated genes included Sult1e1, Gm11974, monocyte to macrophage differentiation associated 2 (Mmd2), Gm14403, and heat shock protein 90 alpha family class A member 1 (Hsp90aa1), which encodes protein Hsp90A that interacts with a number of tumor promoting proteins [127]. BDE-99 also up-regulated zinc finger protein 445 (Zfp445), tumor suppressor sterile alpha motif domain containing 9 like (Samd9l) [128], aldehyde oxidase 1 (Aox1), roundabout guidance receptor 1 (Robo1), and ileal bile acid uptake transporter solute carrier family 10 member 2 (Slc10a2). Conversely, the top 10 most down-regulated genes by BDE-99 in GF mice included C-C motif chemokine ligand 5 (Ccl5), which recruits leukocytes into inflammatory sites [129]; calcium/calmodulin dependent protein kinase II beta (Camk2b), which promotes synapse formation and maintains synaptic plasticity in brain [130]; microtubule associated protein 1 light chain 3 alpha (Map1lc3a), which mediates the interactions between microtubules and cytoskeleton [131]; calneuron 1 or calcium-binding protein (Caln1); carboxylesterase 4A (Ces4a), which is responsible for the hydrolysis of xenobiotics and also involved in fatty acid metabolism [132]; WAP four-disulfide core domain 2 (Wfdc2), which constitutes the core of the protein and functions as protease inhibitor [133]; guanylate cyclase activator 1A (Guca1a), which is involved in the calcium-dependent regulation of guanylate cyclases in the retina [134]; H2-Eb1, Flywch2, and Serpina3c. Ingenuity pathway analysis (IPA) was performed for the genes that were uniquely regulated following BDE-99 exposure in GF mice, and is shown in S8 Fig, which includes pathways related to developmental disorder, gene expression, protein synthesis, cellular assembly and organization, cellular development, and DNA replication, recombination and repair. An example network (gene expression, protein synthesis, cellular assembly and organization, network #2) is visualized in S8 Fig. BDE-99 down-regulated a large number of mitochondrial ribosomal proteins (Mrpl) in livers of GF mice. Mitochondrial ribosomal proteins are responsible for oxidative phosphorylation to produce energy for cell growth, differentiation and development [75]. The down-regulated Mrpl by BDE-99 treatment may lower ATP production and disturb normal cellular functions, which may ultimately lead to cell death. The same finding was also observed in BDE-99 treated CV mice, indicating BDE-99 mediated reduction of Mrpl are independent of gut microbiome.

### Hepatic transcriptome of lncRNAs that were differentially regulated by PBDEs

**Fig 3:**
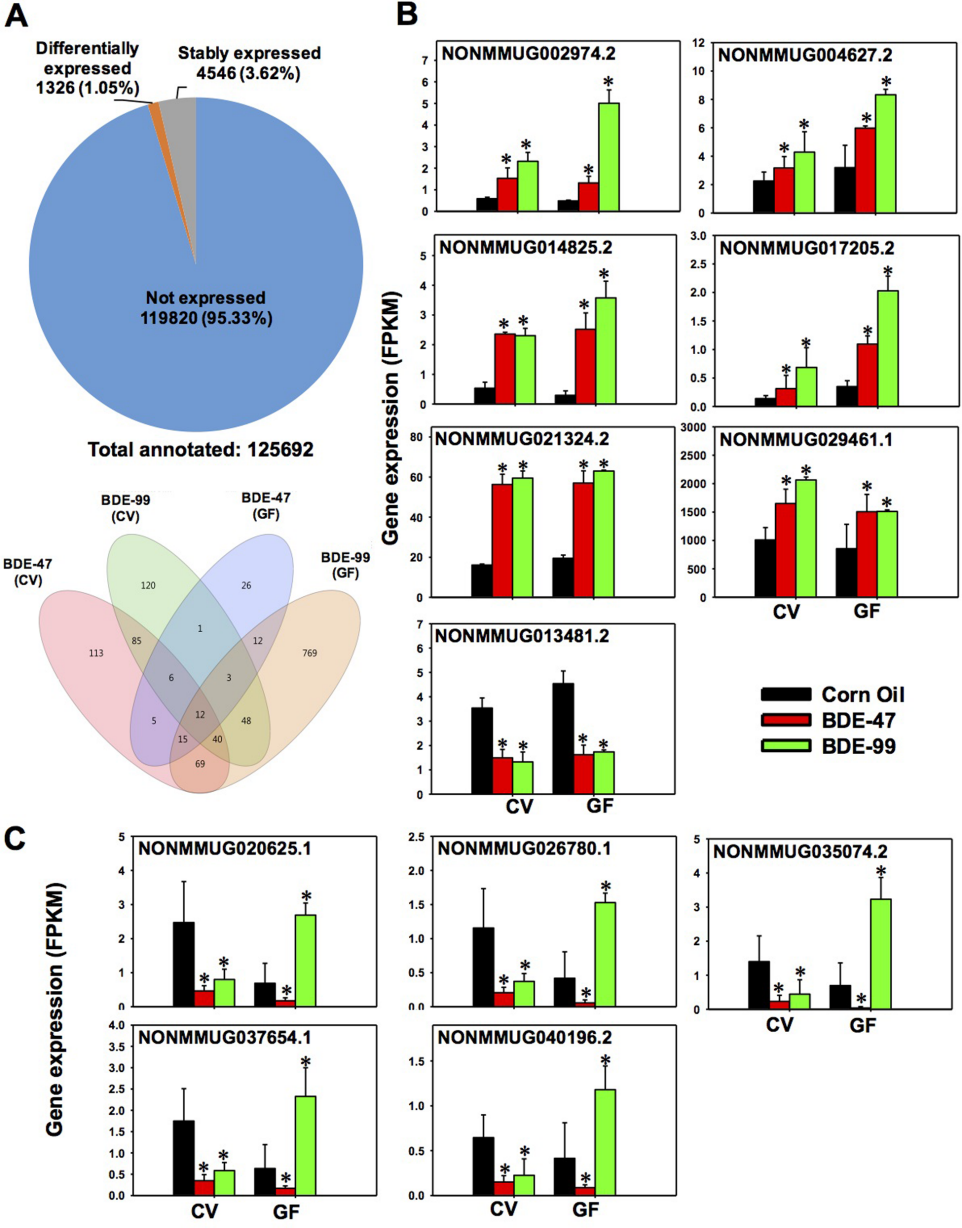
Regulation of long noncoding RNAs (lncRNAs) in livers of CV and GF mice treated with corn oil, BDE-47 (100 μmol/kg), or BDE-99 (100 μmol/kg). **A.** Top: a pie chart showing lncRNAs that were not expressed in any groups (blue), stably expressed in all groups (grey), and differentially expressed by PBDE treatment in at least one of the four comparisons (orange). These four comparisons are: CV_CO (corn oil) vs CV_BDE-47; CV_CO vs CV_BDE-99; GF_CO vs GF_BDE-47; and GF_CO vs GF_BDE-99, FDR adjusted p-value <0.05). Down: a Venn diagram showing the differentially expressed lncRNAs that were commonly or uniquely regulated by PBDEs among the four comparisons as described in Fig 4A. Venn diagram was generated using JMP Genomics. **B-C.** lncRNAs that were differentially regulated by both PBDEs in livers of CV and GF mice. **B.** lncRNAs that were commonly up-regulated or down-regulated by BDE-47 and BDE-99 in livers of both CV and GF mice. **C.** lncRNAs that were consistently down-regulated by BDE-47 in livers of CV and GF mice, but were oppositely regulated in CV (down-regulation) and GF (up-regulation) mice by BDE-99. Asterisks (*) indicate statistically significant differences as compared to vehicle-treated groups of the same enterotypes of mice (Cuffdiff, *p* < 0.05).

As shown in Fig 3A top panel, among the 125,692 annotated lncRNAs, 119,820 lncRNAs were not detected at significant levels in liver of any groups (threshold: average FPKM < 1 in all treatment groups). Among the detected lncRNAs (average FPKM >1 in at least one of the groups), 4,546 lncRNAs were stably expressed across all treatment groups, whereas a total of 1,326 lncRNAs were differentially expressed by PBDEs in livers of CV and GF mice (criteria: *p* < 0.05 in at least one of the 4 comparisons between corn oil (CO) and PBDE-exposed groups of the same enterotype, namely 1) corn oil exposed CV mice (CV_CO) vs. CV_BDE-47, 2) CV_CO vs. CV_BDE-99, 3) corn oil exposed GF mice (GF_CO) vs. GF_BDE-47, and 4) GF_CO vs. GF_BDE-99.

A Venn diagram of these 1,326 lncRNAs revealed a clear separation by PBDE exposure and enterotypes (Fig 3A bottom panel). Regarding the PBDE effect between CV BDE-47 (pink color) and CV BDE-99 (green color), there were 143 lncRNAs commonly regulated by both PBDEs, whereas 202 lncRNAs were uniquely regulated by CV BDE-47, and 172 lncRNAs were uniquely regulated by CV BDE-99. Similarly, between GF BDE-47 (purple color) and GF BDE-99 (orange color), there were 42 lncRNAs commonly regulated by both PBDEs, whereas 38 lncRNAs were uniquely regulated by GF BDE-47, and 926 lncRNAs were uniquely regulated by BDE-99 in livers of GF mice. Regarding the necessity of gut microbiome in modulating PBDE-mediated lncRNAs expression, between CV BDE-47 and GF BDE-47, there were 38 lncRNAs commonly regulated by BDE-47 in both CV and GF mice, whereas 307 lncRNAs were uniquely regulated by BDE-47 in CV mice, and only 42 lncRNAs were uniquely regulated by BDE-47 in GF mice. Between CV BDE-99 and GF BDE-99, there were 103 lncRNAs commonly regulated by BDE-99 in both CV and GF mice. To note, there were many more lncRNAs that were uniquely regulated by BDE-99 in GF mice (865 lncRNAs in total) compared to that in CV mice (212 lncRNAs in total). In summary, both PBDEs and gut microbiome regulated the hepatic transcriptome of lncRNAs in mice, and the lack of gut microbiome appeared to sensitize mouse liver to BDE-99-mediated transcriptional regulation of lncRNAs. This trend (Fig 3A bottom panel) shared a high similarity as compared to the regulation of PCGs (Fig 2A right panel).

Among the 4 comparison groups (CV_CO vs. CV_BDE-47, CV_CO vs. CV_BDE-99, GF_CO vs. GF_BDE-47, and GF_CO vs. GF_BDE-99), there were 12 lncRNAs that were commonly regulated by both BDE-47 and BDE-99 in livers of both CV and GF mice. Further analysis of these 12 lnRNAs revealed 2 distinct patterns (Fig 3B and 3C): pattern 1 included 7 lncRNAs that were consistently up-regulated or consistently down-regulated by both PBDEs in CV and GF mice, including NONMMUG002974.2, NONMMUG004627.2, NONMMUG014825.2, NONMMUG017205.2, NONMMUG021324.2, NONMMUG029461.1, and NONMMUG013481.2 (Fig 3B). Pattern 2 included 5 lncRNAs that were consistently down-regulated by BDE-47 in livers of both CV and GF mice, but were regulated by BDE-99 in a completely opposite manner (Fig 3C), which is the lack of gut microbiome abolished BDE-99-mediated down-regulation of these 5 lncRNAs in CV mice, resulting in a marked up-regulation of these lncRNAs in livers of GF mice.

### Genomic annotation of lncRNAs that were differentially regulated by PBDEs

To test our hypothesis that the differentially regulated lncRNAs by PBDEs and gut microbiome are produced from distinct genomic regions proximal to key regulatory DNA elements of PCGs, we used PAVIS to annotate the genomic locations of differentially regulated lncRNAs relative to their neighboring PCGs (within 5 kb upstream and 1 kb downstream of the neighboring PCGs, which is the recommended setting of the software).

Under control conditions, lack of gut microbiome altered the basal expression of 211 lncRNAs in liver, among which 90 lncRNAs were located within the genomic distance of neighboring PCGs. As shown in S9 Fig, the majority of these lncRNAs were mapped to the intron region (35%) of neighboring PCGs, followed by 3’-UTR (27%), intergenic region (18%), upstream of transcriptional start site (TSS) (7%), downstream of transcriptional termination site (TTS) (6%), exonic region (6%), and 5’-UTR region (1%). Therefore, it is possible that the majority of these differentially lncRNAs are produced as a result of post-transcriptional splicing from the nascent mRNA transcript including intron-excision and shorting of 3’-UTR.

**Fig 4:**
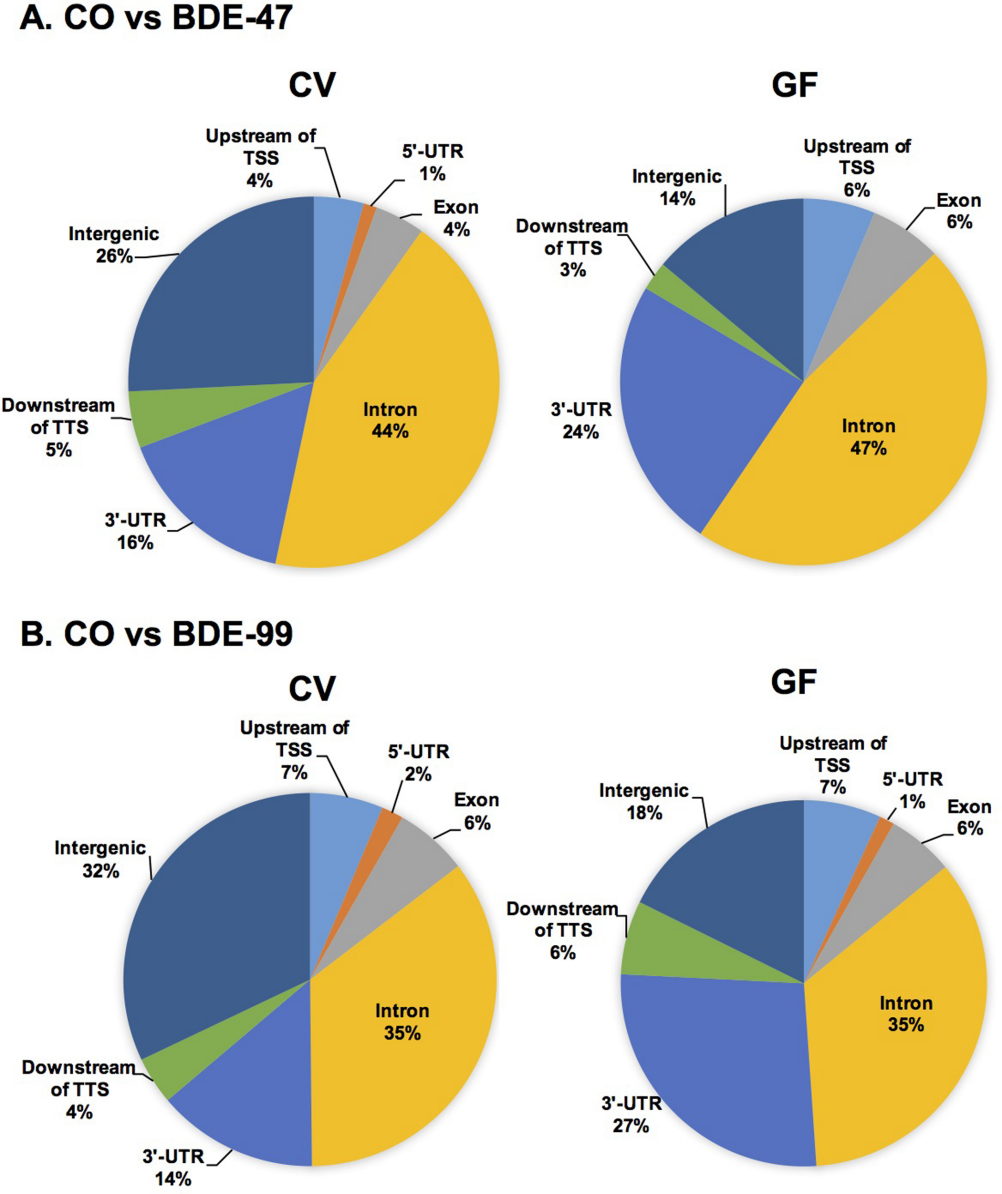
Genomic annotation of differentially regulated lncRNAs relative to the closest PCGs, including up to 5 kb upstream of transcription start site (TSS), intronic, exonic, 5’-untranslated region (UTR), 3’-UTR, and up to 1 kb downstream of transcriptional termination site (TTS). **A.** lncRNAs that were differentially regulated by BDE-47 in livers of CV (left) and GF (right) mice, compared to the vehicle-treated control group of the same enterotypes of mice. **B.** lncRNAs that were differentially regulated by BDE-99 in livers of CV (left) and GF (right) mice, compared to the vehicle-treated control group of the same enterotypes of mice.

Regarding the BDE-47 effect, in livers of CV mice, BDE-47 regulated 345 lncRNAs, among which 255 lncRNAs were located within the genomic distance to their neighboring PCGs. As shown in Fig 4A, the majority of these lncRNAs were mapped to the intron region (44%) of neighboring PCGs, followed by intergenic region (26%), 3’-UTR (16%), downstream of TTS (5%), upstream of TSS (4%), exonic region (4%), and 5’-UTR region (1%). In livers of GF mice, BDE-47 differentially regulated 80 lncRNAs compared to the control GF group, among which 68 lncRNAs were located within the genomic distance of neighboring PCGs. As shown in Fig 4A, similar to data in CV mice, the majority of them were likewise mapped to the intron region of neighboring PCGs (47%), followed by 3’-UTR region (24%), intergenic region (14%), upstream of TSS (6%), exonic region (6%), and downstream of TTS (3%). Interestingly, compared to CV BDE-47 group, there were more differentially regulated lncRNAs mapped to the 3’-UTR (from 16% CV conditions to 24% GF conditions) in GF BDE-47 group, but less lncRNAs mapped to the intergenic region (from 26% CV conditions to 14% GF conditions).

Regarding the BDE-99 effect, in liver of CV mice, BDE-99 differentially regulated 315 lncRNAs, among which 214 lncRNAs were located within the genomic distance to their neighboring PCGs. As shown in Fig 4B, the majority of these lncRNAs were mapped to the intron region (35%) of neighboring PCGs, followed by intergenic region (32%), 3’-UTR (14%), upstream of TSS (7%), exonic region (6%), downstream of TTS (4%), and 5’-UTR region (2%). In livers of GF mice, BDE-99 differentially regulated 968 lncRNAs compared to the control GF group, among which 794 lncRNAs were located within the genomic distance of neighboring PCGs. As shown in Fig 4B, similar to CV conditions, the majority of them were likewise mapped to the intron region of neighboring PCGs (35%), followed by 3’-UTR region (27%), intergenic region (18%), upstream of TSS (7%), exonic region (6%), downstream of TTS (6%), and 5’-UTR region (1%). Similar to the BDE-47 data, compared to BDE-99-treated CV mice, there were more lncRNAs mapped to the 3’-UTR region (from 14% CV conditions to 27% GF conditions), but less lncRNAs mapped to the intergenic region (from 32% CV conditions to 18% GF conditions) in BDE-99-treated GF mice.

In summary, following BDE-exposure, lack of gut microbiome associated with a substantial increase in the proportions of differentially regulated lncRNAs that were mapped to introns of the PCGs, and a corresponding decrease in the proportions that were mapped to intergenic regions. LncRNAs have been reported to function as miRNA decoy in regulating the expression of PCGs via competing with miRNAs for the 3’-UTR binding sites [135, 136]. The increased lncRNAs mapped to 3’-UTR regions in the GF conditions suggested that they may contribute to increased translation efficiency of the neighboring PCGs by preventing miRNA functions.

### lncRNA-PCG pairs that were differentially regulated by PBDEs

To predict the potential functions of the differentially regulated lncRNAs by PBDEs, we paired the differentially regulated lncRNAs with their neighboring PCGs as defined by two criteria: 1) the lncRNA overlaps within 5 kb upstream of TTS and 1 kb downstream of TTS of its closest PCG; 2) both lncRNA and its neighboring PCG are significantly differentially regulated by PBDEs (*p* <0.05). As shown in Table 3, in livers of CV mice, there were 132 lncRNA-PCG pairs differentially regulated by BDE-47 and 130 pairs differentially regulated by BDE-99; whereas in livers of GF mice, there were 19 lncRNA-PCG pairs differentially regulated by BDE-47, and 544 pairs differentially regulated by BDE-99. The gene symbols of the identified lncRNA-PCG pairs from each comparison are present in S3 Table. Pathway analysis was performed using the paired neighboring PCGs to predict the signaling pathways potentially regulated by the neighboring co-regulated lncRNAs.

**Table 3:** Differentially regulated lncRNAs paired with neighboring PCGs by PAVIS. lncRNA-PCG pair was defined as lncRNAs overlapped with or within 5 kb upstream and 1 kb downstream of closest PCGs.

Transcripts	CV_BDE-47	CV_BDE-99	GF_BDE-47	GF_BDE-99
lncRNAs differentially regulated	345	315	80	968
Annotated lncRNAs	255	214	68	794
lncRNA-PCG pairs	132	130	19	544

**Fig 5:**
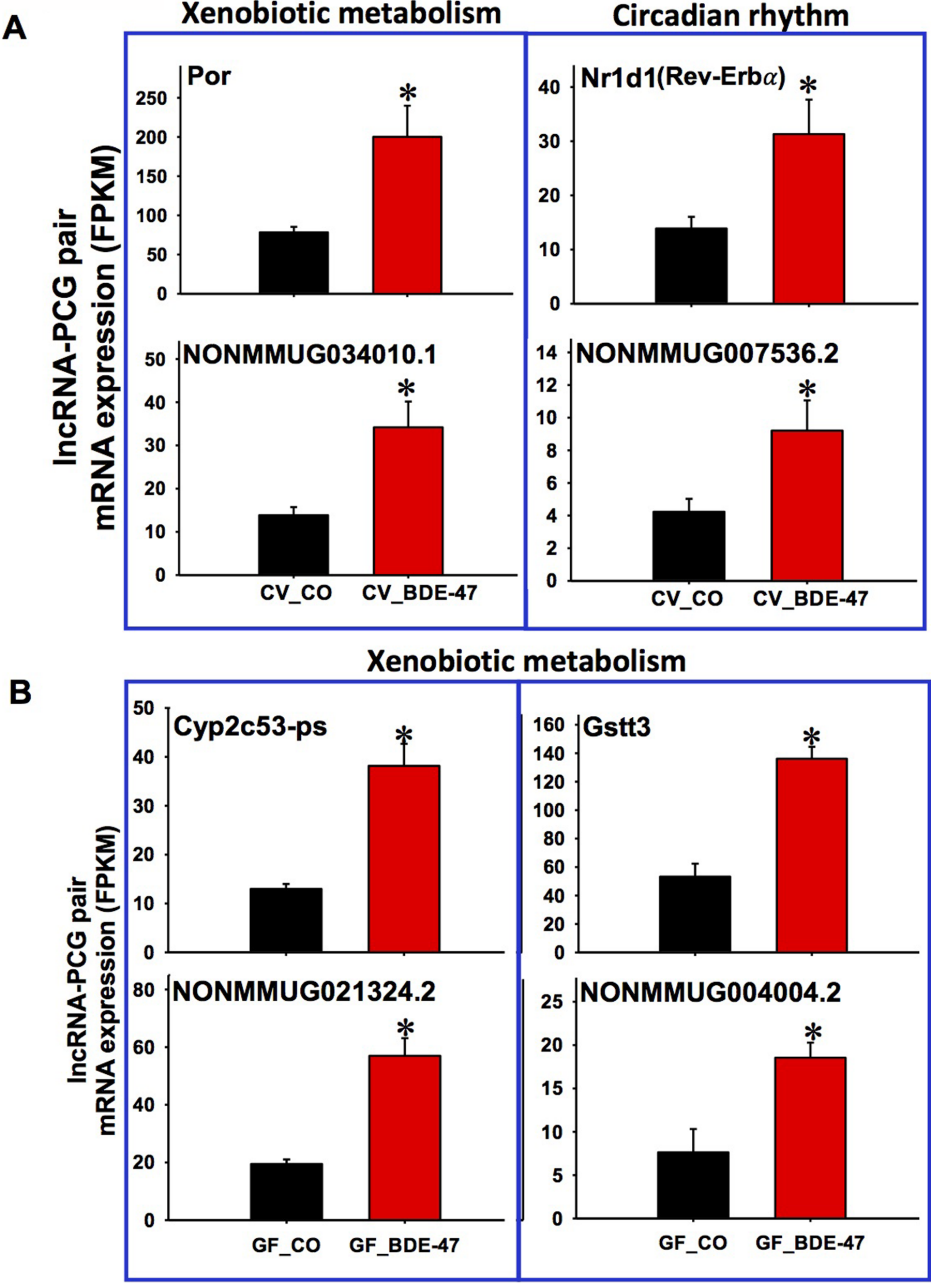
Representative PCG-lncRNA pairs and the related signaling pathways in CV_CO vs. CV_BDE-47 exposed conditions (A) as well as in GF_CO vs. GF_BDE-47 exposed conditions (B). **A.** Xenobiotic metabolism pathway (Por) and circadian rhythm pathway (Nr1d1/Rev-Erbα). **B.** Xenobiotic metabolism pathway (Cyp2c53-ps and Gstt3). Asterisks (*) represent statistically significant differences between the two conditions (Cuffdiff, *p* < 0.05).

As shown in S10 Fig, in livers of CV mice exposed to BDE-47, the major pathways for the differentially regulated PCGs that were paired with lncRNAs include nucleic acid metabolic process, cellular metabolic process, retinol metabolism, and circadian rhythm. Examples of the lncRNA-PCG pairs involved in retinol metabolism and circadian rhythm are shown in S11 and S12 Figs, which describe not only their genomic structures but also the expression of the PCG and the paired lncRNA. For example, the lncRNA NONMMUG030290.1 is located at chromosome 4 and is 2508 bp long. NONMMUG030290.1 is transcribed from the intron region of its neighboring PCG Cyp4a12a (a fatty acid oxidation enzyme) and ends up at its 3’-UTR region (S11A Fig). Both NONMMUG030290.1 and Cyp4a12a were up-regulated by BDE-47 in livers of CV mice (S11 Fig). The up-regulation of NONMMUG030290.1 by BDE-47 in livers of CV mice was further validated by RT-qPCR (S19A Fig). NONMMUG020961.1 is another intronic lncRNA, which is located at chromosome 19 and it is 74,299 bp long. NONMMUG020961.1 and its neighboring PCG Aldh1a1 were both up-regulated by BDE-47 in livers of CV mice (S11A and S11B Fig). The lncRNA NONMMUG032898.1 is located at chromosome 5 with 8.4 kb in length. It is an intronic lnRNA and is transcribed from the intron region of its neighboring PCG Ugt2b1 (a phase II drug metabolizing enzyme involved in glucuronidation) (S11A Fig). NONMMUG032898.1 and Ugt2b1 were both up-regulated by BDE-47 in livers of CV mice (S11B Fig). Cyp4a12a, Aldh1a1 and Ugt2b1 are known involved in the oxidative metabolism and glucuronidation of retinoid substrates [137, 138]. Other co-upregulated lncRNA-PCG pairs include: the lncRNA NONMMUG034010.1, which is transcribed from the intron region of the P450 reductase Por (which modulates the activities of the phase-I oxidative metabolism) and also overlaps with several exon regions of Por (Fig 5A) (validated by RT-qPCR [S19B Fig]); the lncRNA NONMMUG007536.2, which is antisense to Nr1d1 (also known as Rev-Erbα) that is involved in circadian rhythm (Fig 5A) [139].

In livers of GF mice, there were only 19 lnRNA-PCG pairs differentially regulated by BDE-47. For example, NONMMUG021324.2, which is located at chromosome 4, is completely overlapped with the pseudogene Cyp2c53-ps. Both NONMMUG021324.2 and Cyp2c53-ps were up-regulated by BDE-47 in livers of GF mice (Fig 5B). NONMMUG004004.2, which is located at chromosome 10 and is antisense to Gstt3. NONMMUG004004.2 and Gstt3 were also up-regulated by BDE-47 in livers of GF mice (Fig 5B). In addition, several lncRNA-paired PCGs involved in cholesterol and lipid metabolism were down-regulated by BDE-47 in livers of GF mice: NONMMUG029453.1, which is transcribed from the downstream region of Gm2083; NONMMUG013481.2, which is transcribed from the intronic region of Tsc22d1 that is related to cholesterol metabolism [86]; as well as NONMMUG020688.1, which is transcribed from the intronic region of transmembrane 7 superfamily member 2 (Tm7sf2, also known as delta(14)-sterol reductase) that is responsible for sterol biosynthesis [140].

In summary, compared to CV mice, lack of gut microbiota sensitized liver to BDE-99 mediated co-regulation of PCG-lncRNA pairs, and specifically, there appeared to be a decrease in PPARα-mediated lipid metabolism and bile acid homeostasis, but an increase in PXR-mediated xenobiotic oxidation.

**Fig 6:**
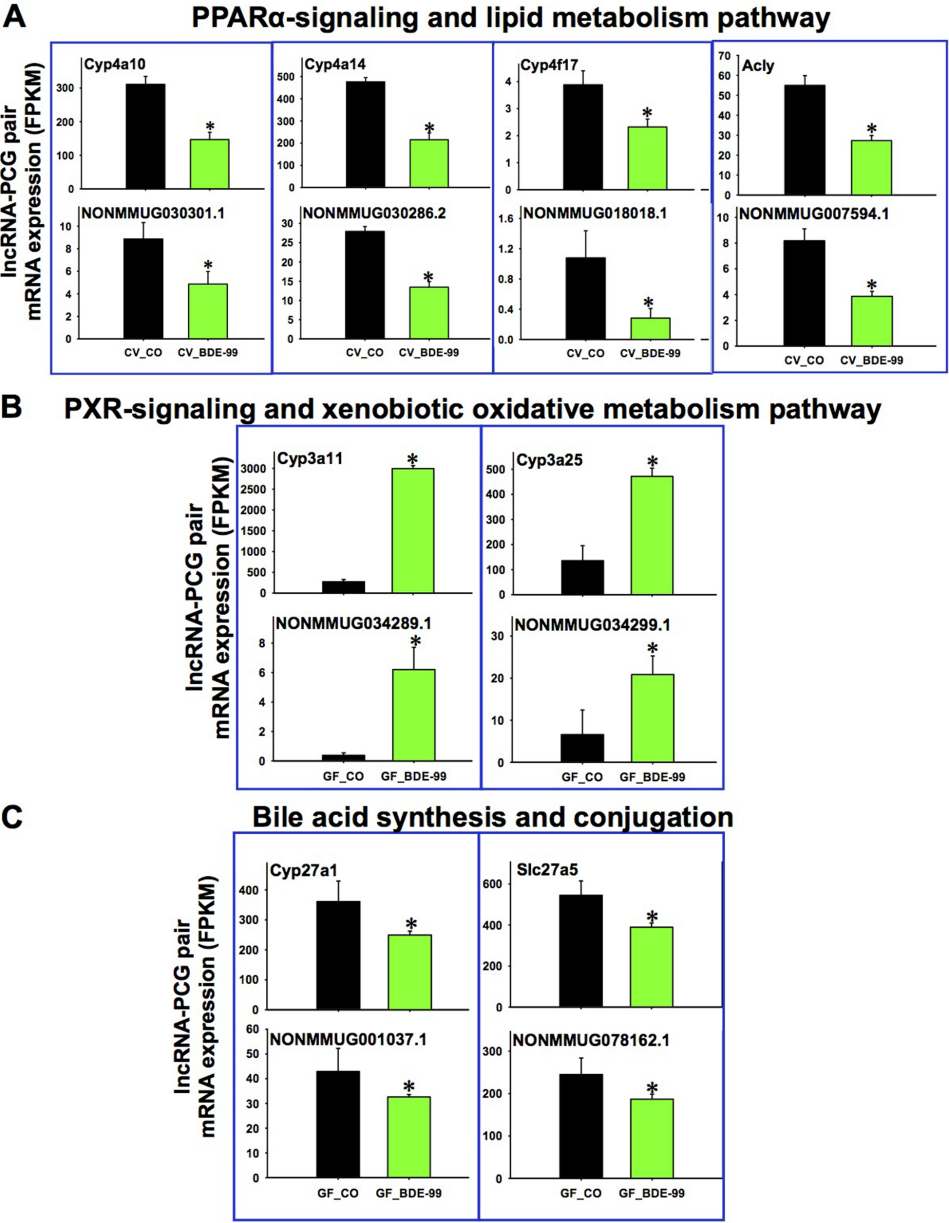
Representative PCG-lncRNA pairs and the related A. PPARα-signaling pathway in CV_CO vs. CV_BDE-99 exposed conditions; **B.** PXR-signaling and xenobiotic oxidative metabolism pathway in GF_CO vs. GF_BDE-99 exposed conditions; and **C.** bile acid synthesis and conjugation pathway in GF_CO vs. GF_BDE-99 exposed conditions. Asterisks (*) represent statistically significant differences between the two conditions (Cuffdiff, *p* < 0.05).

In livers of CV mice, the top pathways for the lnRNA-PCG pairs that were differentially regulated by BDE-99 include metabolic pathway and cellular biosynthetic process (S12 Fig). Examples of the lncRNA-PCG pairs that were differentially regulated by CV BDE-99 are shown in Figs 6A and S13. Interestingly, four PCGs involved in the PPARα-mediated lipid metabolism pathway were consistently down-regulated in livers of BDE-99 exposed CV mice, together with their paired lncRNAs. These down-regulated PPARα-targeted PCGs include the fatty acid oxidation enzymes Cyp4a10, 4a14, and 4f17 [141], as well as ATP citrate liase (Acly) that is involved in the synthesis of acetyl-CoA during lipogenesis and cholesterogenesis. To note, hepatic Acly may also serve as a potential target to treat NAFLD and type II diabetes [142].

In livers of GF mice, three PCGs involved cytochrome P450-mediated phase-I oxidative metabolism were up-regulated (Cyp3a11, Cyp3a25, and Por) by BDE-99, together with their paired lncRNAs (Figs 6B and S13). The lncRNA NONMMUG034299.1 paired with Cyp3a25 was validated by RT-qPCR [S19C Fig]). In contrast, two PCGs involved in bile acid synthesis (Cyp27a1, a bile acid-synthetic enzyme in alternative pathway) and bile acid conjugation (Slc27a5) were down-regulated by BDE-99, together with their paired lncRNAs (Fig 6C). In addition, the histone deacetylase 5 (Hdac5), an epigenetic enzyme involved in removing the histone acetylation mark, transcription repression, cell differentiation, and liver gluconeogenesis [143], was co-down-regulated with its paired lncRNA (S13 Fig). More lncRNA-PCG pairs that were co-up-regulated by BDE-99 in GF conditions are shown in S18 Fig. These paired PCGs are involved in drug metabolism (the phase-II conjugation enzyme Ugt2b36), cholesterol synthesis (Hmgcs1), as well as glutathione synthesis (Gclc) (S18 Fig).

In Summary, compared to CV mice, lack of gut microbiota sensitized liver to BDE-99 mediated co-regulation of PCG-lncRNA pairs, and specifically, there appeared to be a decrease in PPARα-mediated lipid metabolism and bile acid homeostasis, but an increase in PXR-mediated xenobiotic oxidation.

Taken together, the present study has systematically characterized the effect of the interactions between orally exposed PBDEs and gut microbiome on mouse liver transcriptome including both PCGs and lncRNAs. It is the first to demonstrate the necessity of the gut microbiota in modulating the PBDE-mediated differential expression of PCGs and lncRNAs in liver, showing that the lack of gut microbiota sensitizes the liver to greater transcriptomic response to the BDE-99 congener but not to the BDE-47 congener. Both xenobiotic metabolism and intermediary metabolism (such as PPARα-mediated lipid metabolism and bile acid synthesis) are impacted by the interactions between PBDEs and gut microbiota. Unique lncRNA-PCG pairs that are co-differentially regulated by PBDEs and the lack of gut microbiome are identified. These co-regulated lncRNAs may act in concert with transcription factors to promote the formation of PCG transcripts in the same genomic neighborhood to increase their transcription efficiency. In livers of PBDE-treated GF mice, the increased percentages of differentially regulated lncRNAs mapping to the 3’-UTR region may increase the translational efficiency of PCGs by antagonizing the miRNA functions, in response to increased xenobiotic insult and the absence of microbial transformation pathways (such as reduction and hydrolysis) in the intestine of GF mice.

Regarding the regulation of PCGs after oral exposure to PBDEs, BDE-47 and BDE-99 produce both overlapping and unique effects in regulating hepatic gene expression in mice. It has been shown that BDE-47 and BDE-99 are activators of xenobiotic sensors CAR and PXR in rodent livers and human hepatocytes, leading to induced expression of cytochrome P450s [7, 8]. Data from the present study are consistent with previous findings regarding the up-regulation of Cyp2b10, which is the prototypical target gene of CAR, by both PBDEs in livers of CV and GF mice. Moreover, BDE-47 and BDE-99 also up-regulate many genes that are known CAR and PXR targets in mice, such as Cyp2b13, Cyp2c55, Alas1, Akr1b7, and Gstm3 (Table 1) [77, 144, 145]. Among them, Alas1 encodes 5-aminolevulinate synthase, which is the rate-limiting enzyme in heme biosynthesis [145, 146]. Up-regulation of Alas1 by PBDEs may increase the synthetic rate of heme, which likely contributes to the increased production of heme-containing proteins like Cyps to further facilitate their enzymatic function. Akr1b7 is important for the detoxification of lipid peroxidation [77]. Interestingly, both Alas1 and Akr1b7 are also target genes of bile acid-activated nuclear receptor farnesoid X receptor (FXR), which regulates lipid and glucose homeostasis [79, 147]. Besides xenobiotic metabolism, the activation of CAR and PXR have been linked to diverse physiological pathways, including lipid metabolism, glucose homeostasis, inflammation, and hepatogenesis [148, 149]; all these pathways were highly regulated by PBDEs. PBDEs may also impact bile acid synthesis and fatty acid oxidation since PBDEs also up-regulate Cyp7a1 and Cyp7b1 involved in bile acid synthesis, but down-regulate Cyp4a family members involved in lipid metabolism in CV mice. Another interesting finding is that the absence of gut microbiota potentiated BDE-47-mediated up-regulation of many Gsts, which protect cells from oxidative stress, whereas potentiated BDE-99-mediated down-regulation of a large number of mitochondrial ribosomal proteins that are essential for energy production, suggesting that the livers of GF mice are more vulnerable to oxidative stress and mitochondria damage following additional insult, since gut microbiota is responsible for the majority of reduction reactions of the whole organism [150, 151].

Prior to our study, lncRNAs were increasingly recognized as key regulators of human diseases and served as novel biomarkers of environmental exposure [152]; however, there was no information regarding the regulation of hepatic lncRNAs by PBDEs and gut microbiome, which are two key players in xenobiotic biotransformation in liver [7, 8, 11, 12]. The present study has filled this critical knowledge gap and demonstrates that lncRNAs are profoundly altered by both PBDEs and the lack of gut microbiome in mouse livers, and this suggests that the differentially regulated lncRNAs may also play important roles in PBDE-mediated toxicity.

Importantly, the present study has unveiled the potential interactions between lncRNAs and PCGs by identifying lncRNA-PCG pairs that were uniquely regulated by different PBDE congeners and by the absence of gut microbiome. The lncRNAs paired with PCGs in the same neighborhood may have a greater opportunity to interact with transcription factors that regulate transcription and alternative splicing, possibly preventing miRNA binding to the 3’-UTR of these PCGs simply due to its “geological advantage”. It has been demonstrated that lncRNAs can regulate protein-coding genes when they reside on the opposite strand to the gene [153, 154]. The molecular mechanisms for lncRNAs in regulating gene expression are mainly dependent on their ability to tether with proteins like transcription factors or chromatin modifiers, to either activate or repress their activity in transcription [155]. For example, in CV mice, NONMMUG034010.1, which overlaps with Por, is up-regulated by both BDE-47 and BDE-99 in the same pattern as Por (Figs 5 and S10). NONMMUG030301.1, which is transcribed from the upstream of Cyp4a10 in the same direction, is down-regulated by BDE-99 as Cyp4a10. Whereas NONMMUG030286.2, which is antisense and overlaps with Cyp4a14, is also down-regulated by BDE-99 as Cyp4a14 (Fig 6A). In addition, NONMMUG034289.1, which is antisense and overlapping with Cyp3a11, is markedly up-regulated by BDE-99 as Cyp3a11 in GF mice (Fig 6B). Further studies such as RNA-immunoprecipitation assays are required to elucidate the functions of the differentially regulated lncRNAs by PBDEs and their impact on the related signaling pathways such as metabolic process and lipid metabolism.

Although PBDEs as a class of persistent environmental chemicals are generally thought to display many similar features, such as receptor interaction and toxicities, our study showed that the regulation of PCGs and lncRNAs is PBDE congener-specific, and such regulation is further modified by the absence of gut microbiome. GF mice are more responsive to BDE-99 but not BDE-47 induced changes in gene expression, as indicated by the approximately 3 to 4-fold greater number of responsive lncRNAs and PCGs in GF mice than in CV mice, and by the larger magnitude of gene responses in GF mice after BDE-99 exposure. For BDE-47, however there are many more lncRNAs and PCGs differentially regulated in CV mice than that in GF mice, indicating that the BDE-47-mediated effects on hepatic gene expression are more gut microbiome dependent. Pathway analysis of the neighboring PCGs indicates that BDE-47 in CV mice mainly affects genes involved in nucleic acid metabolic process, cellular metabolic process, retinol metabolism, and circadian rhythm; whereas BDE-99 in CV mice affects metabolic pathway and cellular biosynthetic process. The livers of GF mice are more resistant to BDE-47-mediated effects since less than 100 lncRNAs were differentially expressed. Conversely, there are 968 lncRNAs differentially regulated by BDE-99 in GF mice, with 544 lncRNAs located closely with nearby PCGs. Pathway analysis indicates that in the livers of GF mice, BDE-99 affects diverse biological processes, such as PPARα-signaling and metabolic processes.

The mechanisms of gut microbiome-mediated regulation of PBDE metabolism and the subsequent regulation of hepatic gene expression is not completely understood. Studies have found that the enterohepatic circulation of PBDEs is affected by variation in gut microbial populations, leading to increased toxicity and residence time in the body [156]. Higher levels of hydroxylated BDE-99 metabolites compared to BDE-47 metabolites have been detected in the liver [31]. Therefore, in addition to the transcriptional factors that locate in the liver, the gut microbiota and gut-derived PBDE metabolites may function together to further modify the transcriptome expression in the liver. Because the sequencing libraries in the present study were constructed from poly-A tail selected RNAs, the detected lncRNAs are mainly polyadenylated, which may miss 40% of lncRNA transcripts that are nonpolyadenylated [157]. Future studies using whole transcriptome sequencing, which can capture all forms of lncRNAs, will provide more comprehensive information on the transcriptional mechanisms of lncRNAs and the co-regulated protein coding genes.

In addition to the hepatic effect, as an alternative mechanism, it is possible that the brain may play an important role in modulating the gut-liver axis and the subsequent changes in the hepatic transcriptome. Importantly, PBDEs are neurotoxicant and may directly impact brain signaling [158, 159]. Gut microbiome may secrete distinct neuro-active microbial metabolites such as short chain fatty acids and secondary bile acids, which may enter the systemic circulation to modulate cognitive functions in brain [160–162]. In addition, we do acknowledge that the interactions between gut and liver are likely bi-directional; in that changes in hepatic transcriptome in GF conditions may in turn modulate brain-gut signaling pathways. Future studies are needed to further elucidate this regulatory network and the underlying “remote-sensing” mechanisms.

Oral and nasal microbiomes are also important in toxicological responses in general and especially regarding dietary changes (oral) and air pollution (nasal), considering that the majority of the microbiome is present in the intestine, and the route of exposure in our study was oral gavage, which bypassed the oral and nasal passages, we think that the lack of microbiome (i.e. the germ free mouse model) will have a major impact on the gut, and a relatively minor impact in oral and nasal passages. Therefore, the predominant effect observed in germ free mice should be due to lack of gut microbiome.

We have recorded the body weight daily during the PBDE administration and we have observed no apparent weight gain or change in appetite. Therefore, the changes observed may not be primarily due to intermediary metabolism parameters such as body weight or appetite. However, as we reported previously, there was a basal increase in the expression of multiple Cyp2c gene isoforms in livers of GF mice [31, 163]; and this coincides with increased major hydroxylated metabolite of BDE-47 [31], which is considered more toxic than the parent compound. Therefore, the basal differences in xenobiotic biotransformation related gene expression may at least partially contribute to the changes in PCG-lncRNA gene expression.

Basal differences between corn oil treated GF mice and corn oil treated CV mice were observed as shown in described in S9 Fig and S3 Table (tabs entitled “CVCO_GFCO_PCGs” and “CVCO_GFCO_lncRNAs”). The basal difference is likely due to lack of microbial metabolism of endogenous compounds such as bile acids, amino acids, and complex carbohydrates, which lead to lack of distinct microbial metabolites to interact with the host receptors. For example, using targeted metabolomics, we have demonstrated that lack of gut microbiome leads to a marked decrease in secondary bile acids such as lithocholic acid and the tryptophan microbial metabolite indole-3-propionic acid (submitted manuscript, data not shown). Both of these metabolites have been shown to serve as activators of the host hepatic xenobiotic-sensing nuclear receptor pregnane X receptor (PXR). Using ChIP-qPCR, we have demonstrated that in livers of GF mice, there was reduced PXR-DNA binding to the targeted protein-coding gene Cyp3a locus, which encodes the major phase-I drug-metabolizing enzyme in liver. This corresponds to reduced Cyp3a mRNA, protein, and enzyme activities. Whereas exposing the GF mice to feces from CV restored PXR-binding and normalized Cyp3a mRNA, protein, and enzyme activities [164]. Interestingly, the reduction in Cyp3a gene expression also coincided with the neighboring lncRNAs (S3 Table), suggesting that lack of microbial metabolites mediated host PXR binding also impacts lncRNA gene expression. Regarding the response to corn oil exposure, because corn oil contains various types of lipids that may require microbial biotransformation, lack of gut microbiome is expected to increase the workload on the hepatic host receptor signaling (such as the lipid-sensing nuclear receptor PPARα signaling). Indeed, we have demonstrated that PPARα-pathway was enhanced in liver of GF mice, evidenced by increased target gene expression and activity, as well as increased PPARα-binding to genomic DNA; whereas conventionalization of GF mice by introducing exogenous bacteria suppressed PPARα in liver [164]. However, because corn oil is the vehicle to dissolve PBDEs, even though the basal differences are observed, for a well-controlled experimental design we need to use the corn oil exposed groups as controls. This has been discussed in our revised manuscript. The remote-sensing mechanisms between gut microbial metabolites and liver response are discussed in detail in our recent review article [10].

In conclusion, the present study unveils the distinct role of gut microbiome in modulating PBDE-mediated gene expression in liver at a transcriptome scale, and for the first time discovers a large number of novel lncRNAs dysregulated by PBDEs, along with their neighboring PCGs. Studies of the regulation and interaction of differentially regulated lncRNAs and PCGs will provide more clues to the mechanisms of PBDEs-mediated effects in diverse biological processes. Moreover, the present study has laid the foundation for future investigations regarding the mechanisms of PBDE-mediated toxicity and molecular biomarkers for PBDE exposure. Further work will be required to validate the direct binding of lncRNAs to the targeted PCGs that share a similar or opposite expression pattern, and determine the translation efficiency of the targeted PCGs using proteomics to unveil the functional impact of lncRNAs on the PCG protein expression.

## Supporting information

S1 Fig Top network of PCGs that were differentially regulated by BDE-47 in livers of CV mice compared to corn oil-treated CV group as analyzed by Ingenuity Pathway Analysis (IPA, *p*<0.05). (TIFF)

S2 Fig Top network of PCGs that were differentially regulated by BDE-99 in livers of CV mice compared to corn oil-treated CV group as analyzed by Ingenuity Pathway Analysis (IPA, p<0.05). (TIFF)

S3 Fig Top network of PCGs that were differentially regulated by BDE-47 in livers of GF mice compared to corn oil-treated GF group as analyzed by Ingenuity Pathway Analysis (IPA, *p*<0.05). (TIFF)

S4 Fig Top network of PCGs that were differentially regulated by BDE-99 in livers of GF mice compared to corn oil-treated GF group as analyzed by Ingenuity Pathway Analysis (IPA, *p*<0.05). (TIFF)

S5 Fig KEGG pathway of PCGs that were commonly regulated by both BDE- 47 and BDE-99 in livers of CV and GF mice as analyzed by STRING Analysis (p<0.05). (TIFF)

S6 Fig Top network of PCGs that were uniquely regulated by BDE-47 in livers of CV mice compared to corn oil-treated CV group as analyzed by Ingenuity Pathway Analysis (IPA, p<0.05). (TIFF)

S7 Fig Top networks of PCGs that were uniquely regulated by BDE-99 in livers of CV mice compared to corn oil-treated CV group as analyzed by Ingenuity Pathway Analysis (IPA, *p*<0.05). (TIFF)

S8 Fig Top network of PCGs that were uniquely regulated by BDE-99 in livers of GF mice compared to corn oil-treated GF group as analyzed by Ingenuity Pathway Analysis (IPA, *p*<0.05). (TIFF)

S9 Fig Genomic annotations of lncRNAs that were differentially regulated between control CV and control GF mice (*p*<0.05) in relative to the closest PCGs. (TIFF)

S10 Fig Pathway analysis of lncRNA-PCG pairs that were differentially regulated by BDE-47 in livers of CV mice compared to corn oil-treated CV group. The lncRNA-PCG pairs between CV_CO and CV_BDE-47 were subjected to STRING analysis using the default settings. The top network is shown.
(TIFF)

S11 Fig Genomic location (**A**) and gene expression (**B**) of lncRNA-PCG pairs that were differentially regulated by BDE-47 in livers of CV mice compared to corn oil-treated CV mice. Drug-metabolizing enzymes Cyp4a12a and aldehyde dehydrogenase (Aldh) 1a1 (phase I), as well as UDP-glucuronosyltransferase (Ugt) 2b1 (phase II) are shown. Expression of lncRNAs and paired PCGs were plotted using mean FPKM ± S.E. Asterisks (*) indicate statistically significant differences as compared to vehicle-treated groups of the same enterotypes of mice (*p* < 0.05).
(TIFF)

S12 Fig Pathway analysis of lncRNA-PCG pairs that were differentially regulated by BDE-99 in livers of CV mice compared to corn oil-treated CV group. The lncRNA-PCG pairs between CV_CO and CV_BDE-99 were subjected to STRING analysis using the default settings. The top network is shown.
(TIFF)

S13 Fig Genomic location (**A**) and gene expression (**B**) of lncRNA-PCG pairs lncRNA-PCG pairs that were differentially regulated by BDE-99 in livers of CV mice compared to corn oil-treated CV group. The epigenetic enzyme histone deacetylase (Hdac) 5 and P450-reductase (Por) are shown.
(TIFF)

S14 Fig Genomic location (**A**) and gene expression (**B**) of lncRNA-PCG pairs lncRNA-PCG pairs differentially regulated by BDE-47 in livers of GF mice (p<0.05) compared to vehicle-treated GF group. Major urinary protein (Gm2083) and two cholesterol metabolism-related genes, namely transforming growth factor beta-stimulated clone 22 homolog (Tsc22d1) and transmembrane 7 superfamily member 2 (Tm7sf2, also known as delta (14)-sterol reductase) are shown here.
(TIFF)

S15 Fig KEGG pathways of lncRNA-PCG pairs that were differentially regulated by BDE-99 in livers of GF mice compared to corn oil-treated GF group (*p*<0.05). (TIFF)

S16 Fig Genomic location (**A**) and gene expression (**B**) of lncRNA-PCG pairs lncRNA-PCG pairs differentially regulated by BDE-99 in livers of GF mice compared to corn oil-treated GF group. Fatty acid desaturase 2 (Fads2), glycoprotein (Cd36), and apolipoprotein (Apo)a1 are shown here.
(TIFF)

S17 Fig Genomic location (**A**) and gene expression (**B**) of lncRNA-PCG pairs lncRNA-PCG pairs differentially regulated by BDE-99 in livers of GF mice compared to corn oil-treated GF group (*p*<0.05). Data were analyzed using STRING Analysis. Apolipoprotein A2 (Apoa2), fatty acid binding protein (Fabp5), and acetyl- CoA acyltransferase 1(Acaa1b) are shown here.
(TIFF)

S18 Fig Genomic location (**A**) and gene expression (**B**) of lncRNA-PCG pairs lncRNA-PCG pairs differentially regulated by BDE-99 in livers of GF mice compared to corn oil-treated GF group (*p*<0.05). The phase II drug metabolizing enzyme UDP-glucuronosyltransferase (Ugt) 2b36, 3-hydroxymethylglutaryl-CoA synthase (Hmgcs1), and Glutathione-cysteine ligase (Gclc), which is the rate-limiting enzyme of glutathione synthesis against oxidative stress, are shown here.
(TIFF)

S19 Fig RT-qPCR validation of selected lncRNAs that paired with important PCGs that are involved in important hepatic metabolic pathways such as lipid metabolism and xenobiotic biotransformation. **A.** Expression of the lncRNA G030290.1 (paired with the fatty acid metabolizing enzyme Cyp4a12a, S11 Fig). **B.** Expression of the lncRNA G034010.1 (paired with the P450 reductase Por, Fig 5). C. Expression of the lncRNA G034299.1 (paired with Cyp3a25, Fig 6B). Data are expressed as % of the house-keeping gene Gapdh. Asterisks represent statistically significant differences as compared to the vehicle-treated group (*p*<0.05).
(TIFF)

S1 Table Mapping statistics of RNA-Seq reads. (PDF)

S2 Table Top 15 networks for PCGs that were differentially regulated by PBDEs in livers of CV and GF mice compared to corn oil-treated control group of the same enterotype of mice. The network in red color was shown in Supplemental Figures.
(PDF)

S3 Table lncRNA-PCG pairs in each comparison (CV BDE-47 vs. CV CO; CV BDE-99 vs. CV CO; GF BDE-47 vs. GF CO; GF BDE-99 vs. GF CO; CV CO vs. GF CO). (XLSX)
