# Supplementary figures and images for: Regulation of protein-coding gene and long noncoding RNA pairs in liver of conventional and germ-free mice following oral PBDE exposure
Source: PLoS One. 2018 Aug 1;13(8):e0201387. doi: 10.1371/journal.pone.0201387 (PMC6070246; doi:10.1371/journal.pone.0201387)

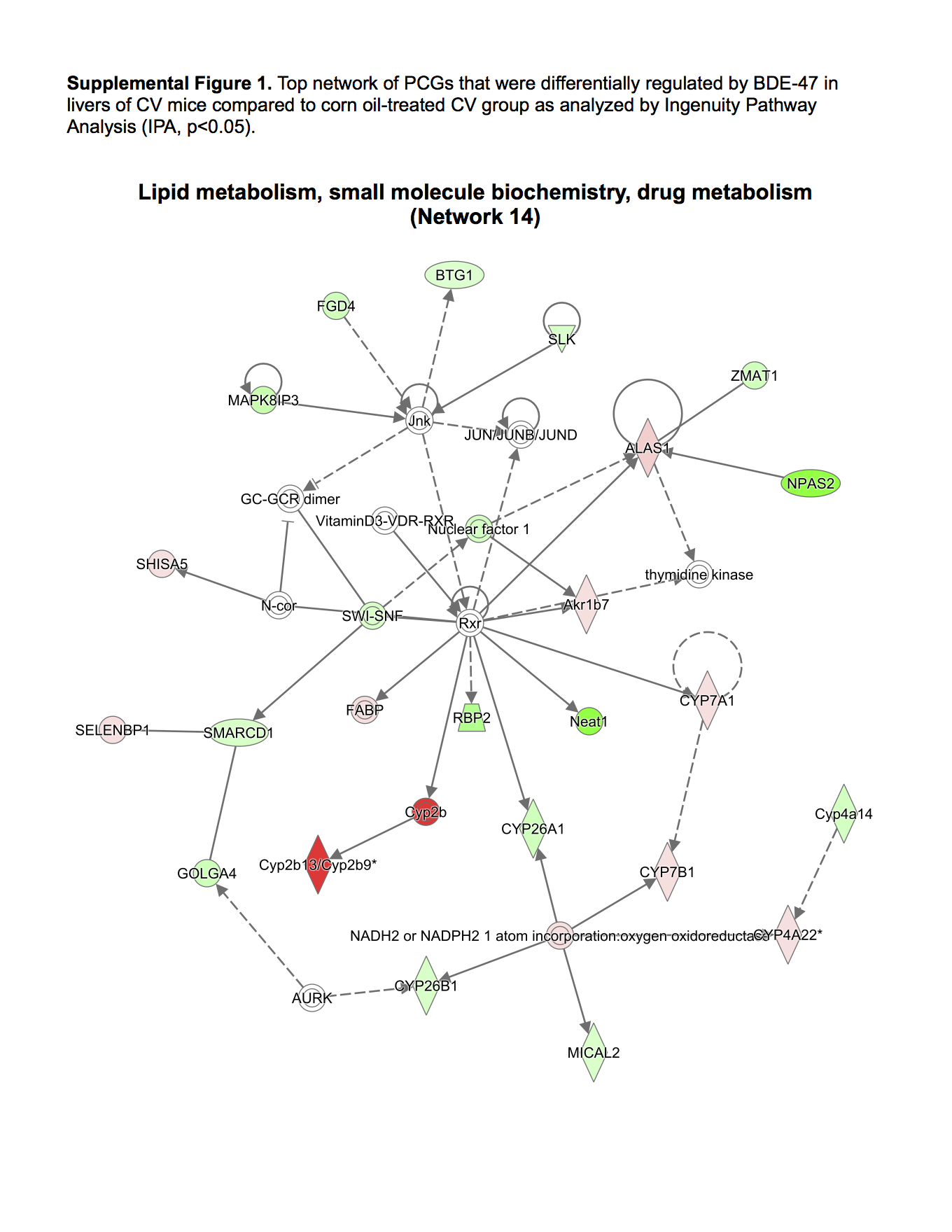

Supplement: S1 Fig — (TIFF) [file pone.0201387.s001.tiff]

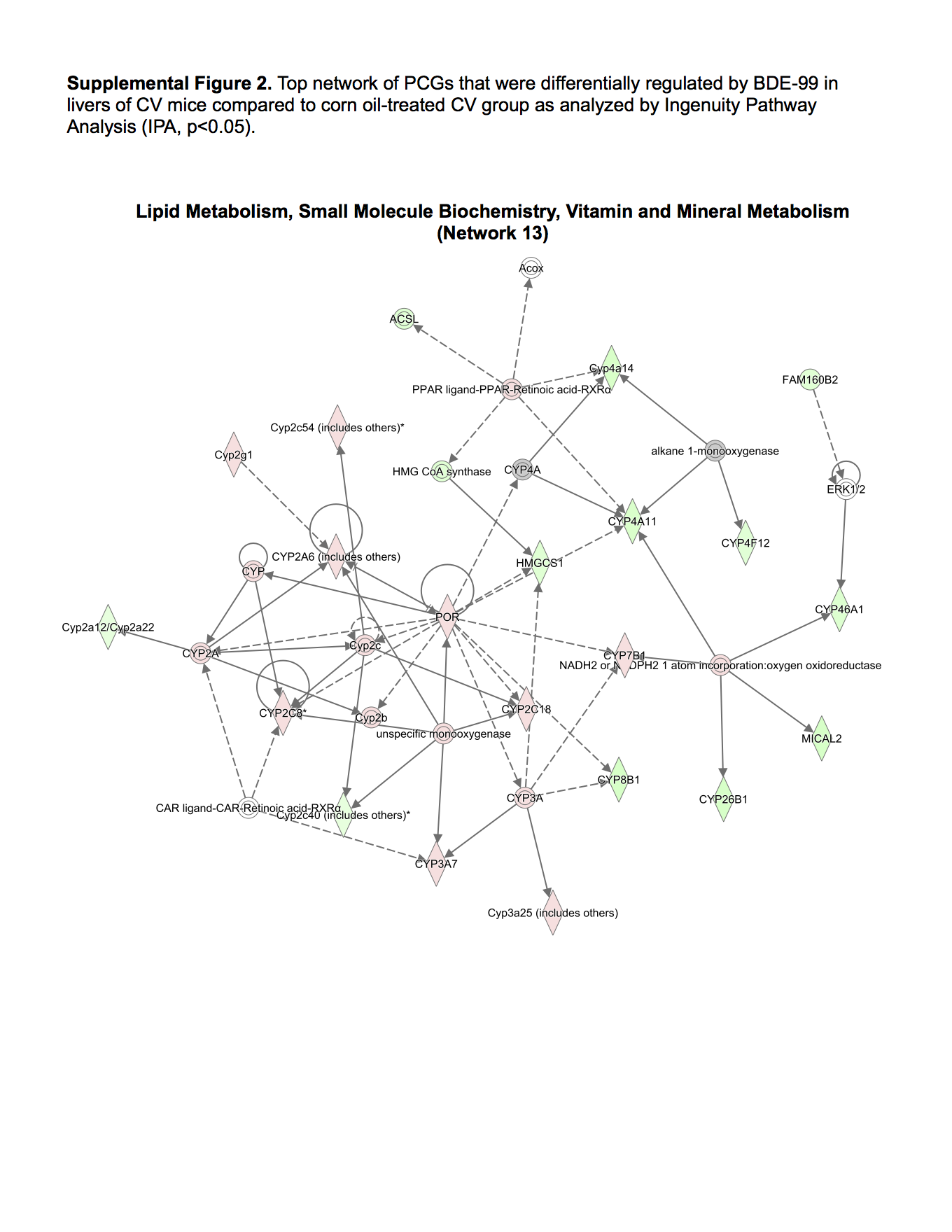

Supplement: S2 Fig — (TIFF) [file pone.0201387.s002.tiff]

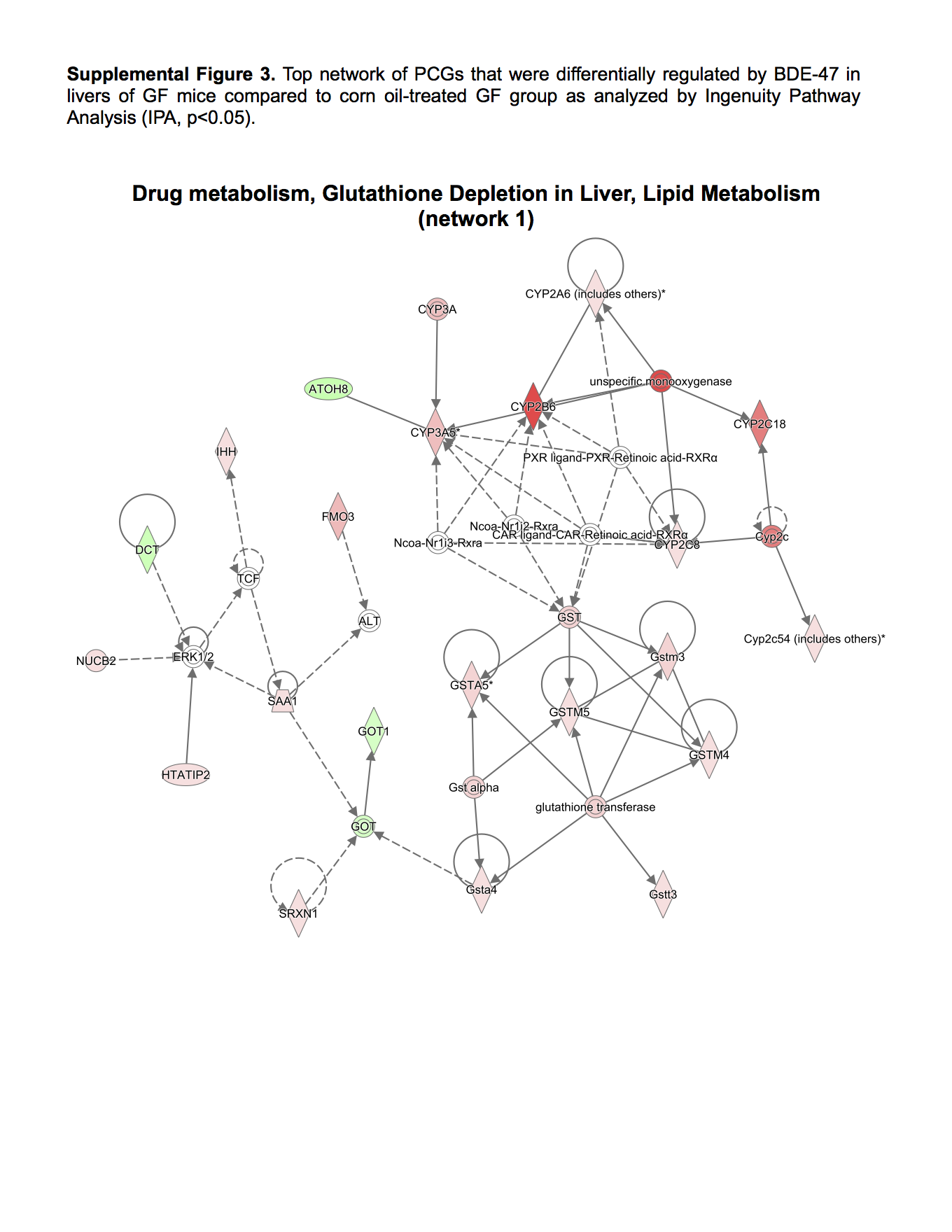

Supplement: S3 Fig — (TIFF) [file pone.0201387.s003.tiff]

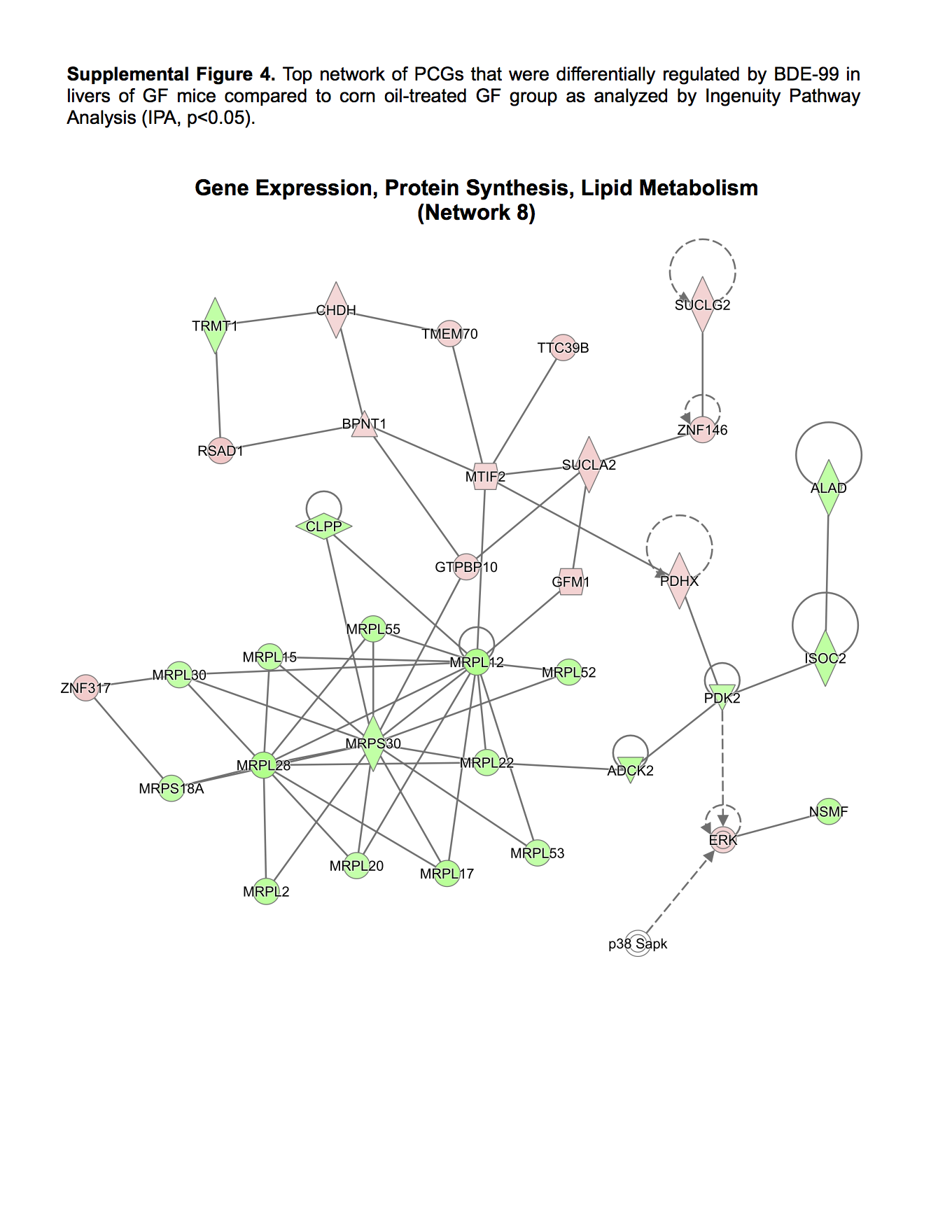

Supplement: S4 Fig — (TIFF) [file pone.0201387.s004.tiff]

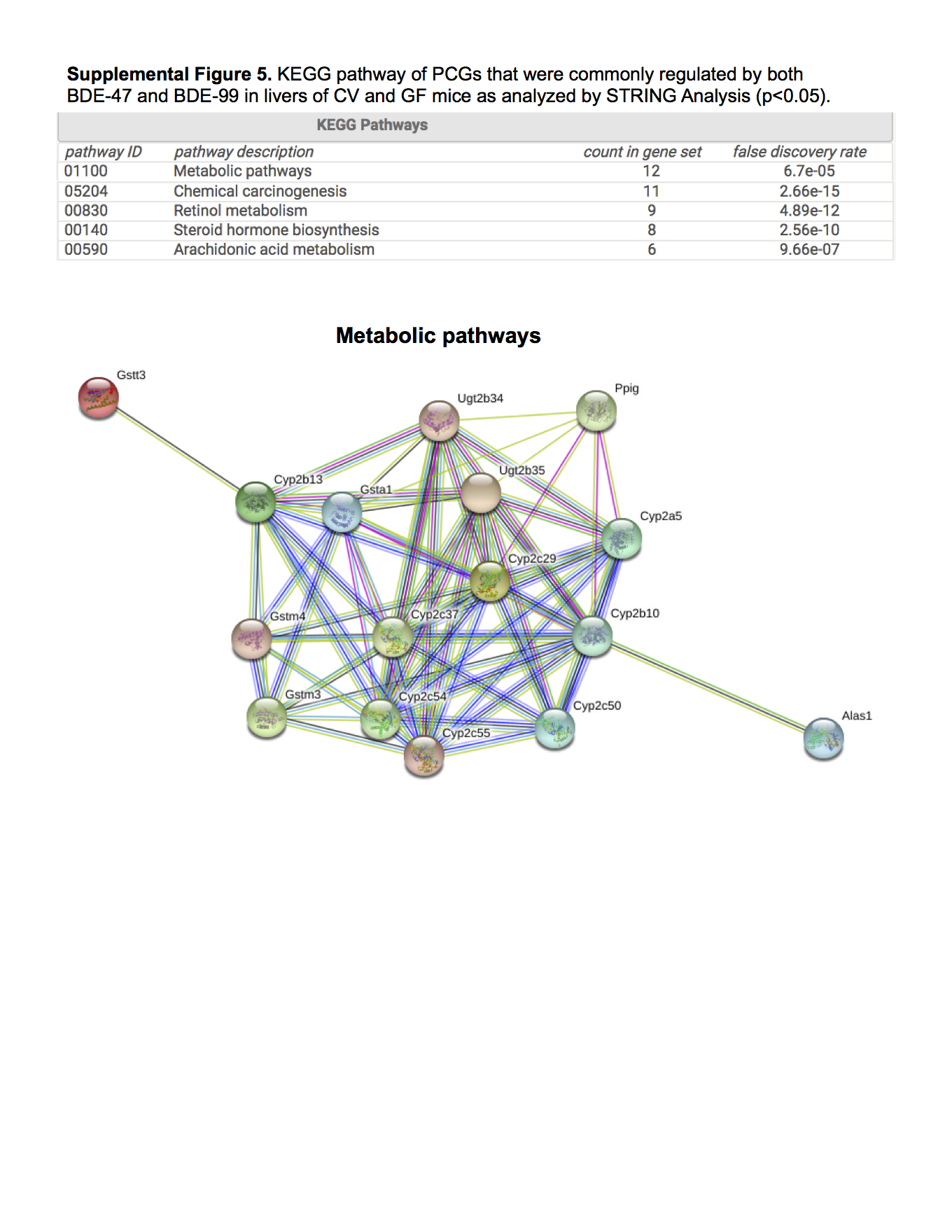

Supplement: S5 Fig — (TIFF) [file pone.0201387.s005.tiff]

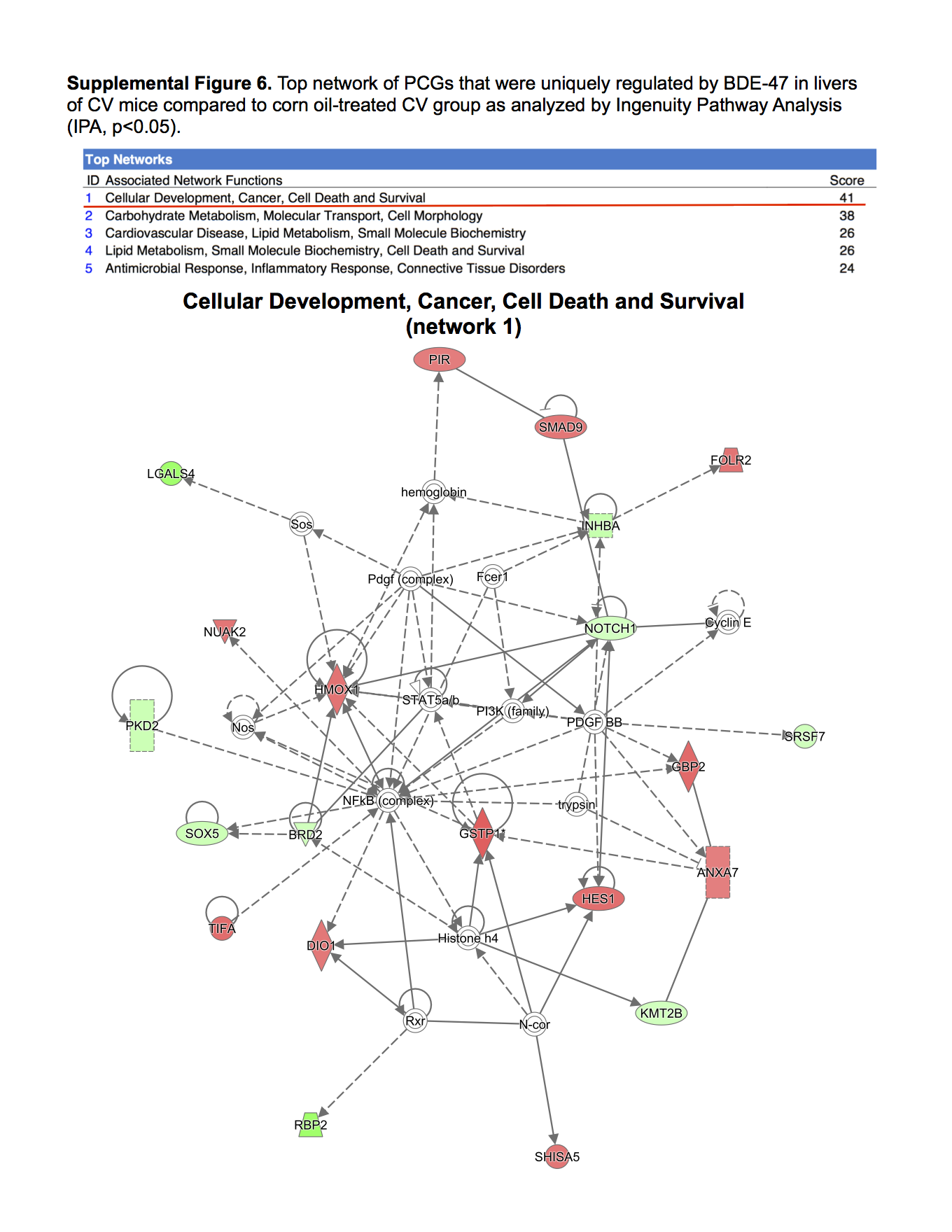

Supplement: S6 Fig — (TIFF) [file pone.0201387.s006.tiff]

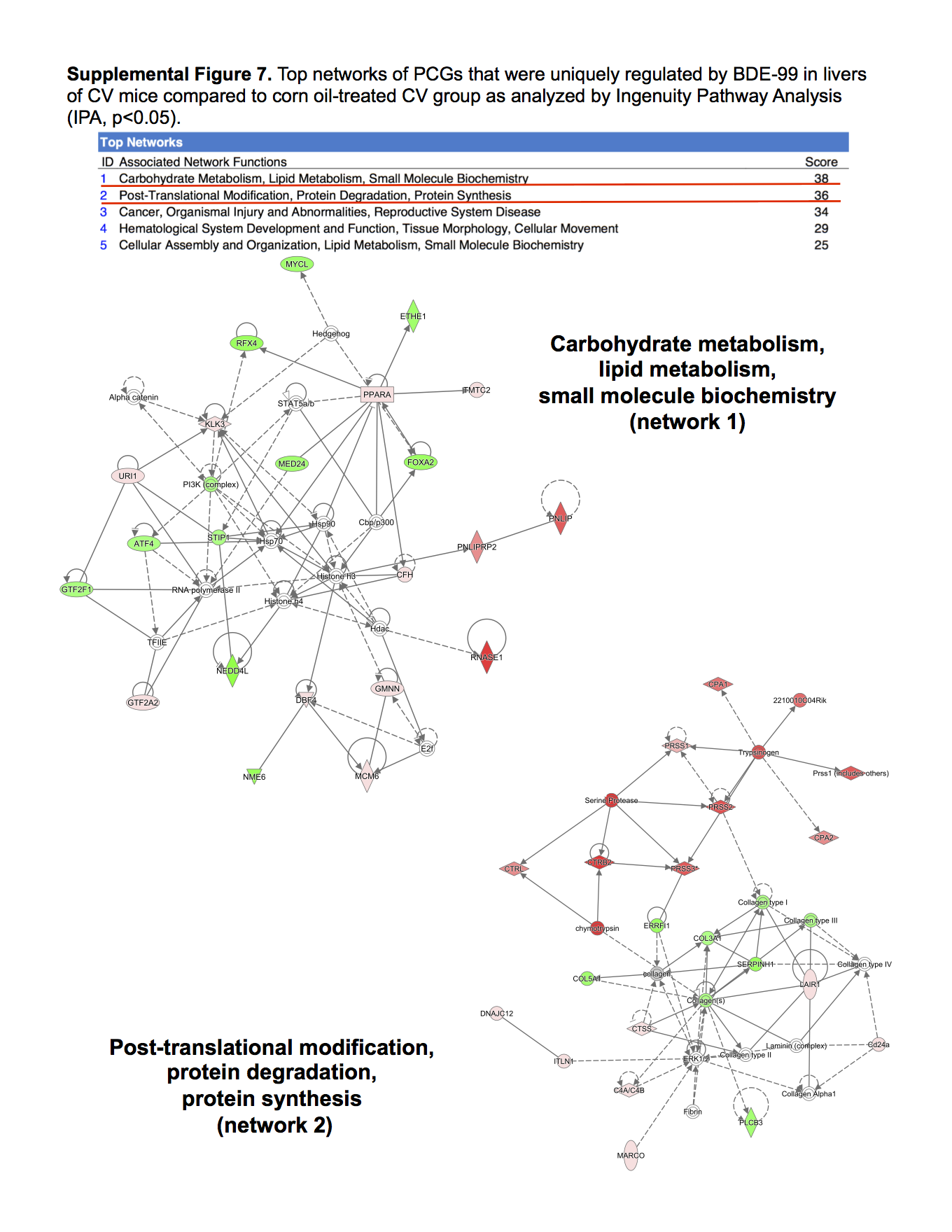

Supplement: S7 Fig — (TIFF) [file pone.0201387.s007.tiff]

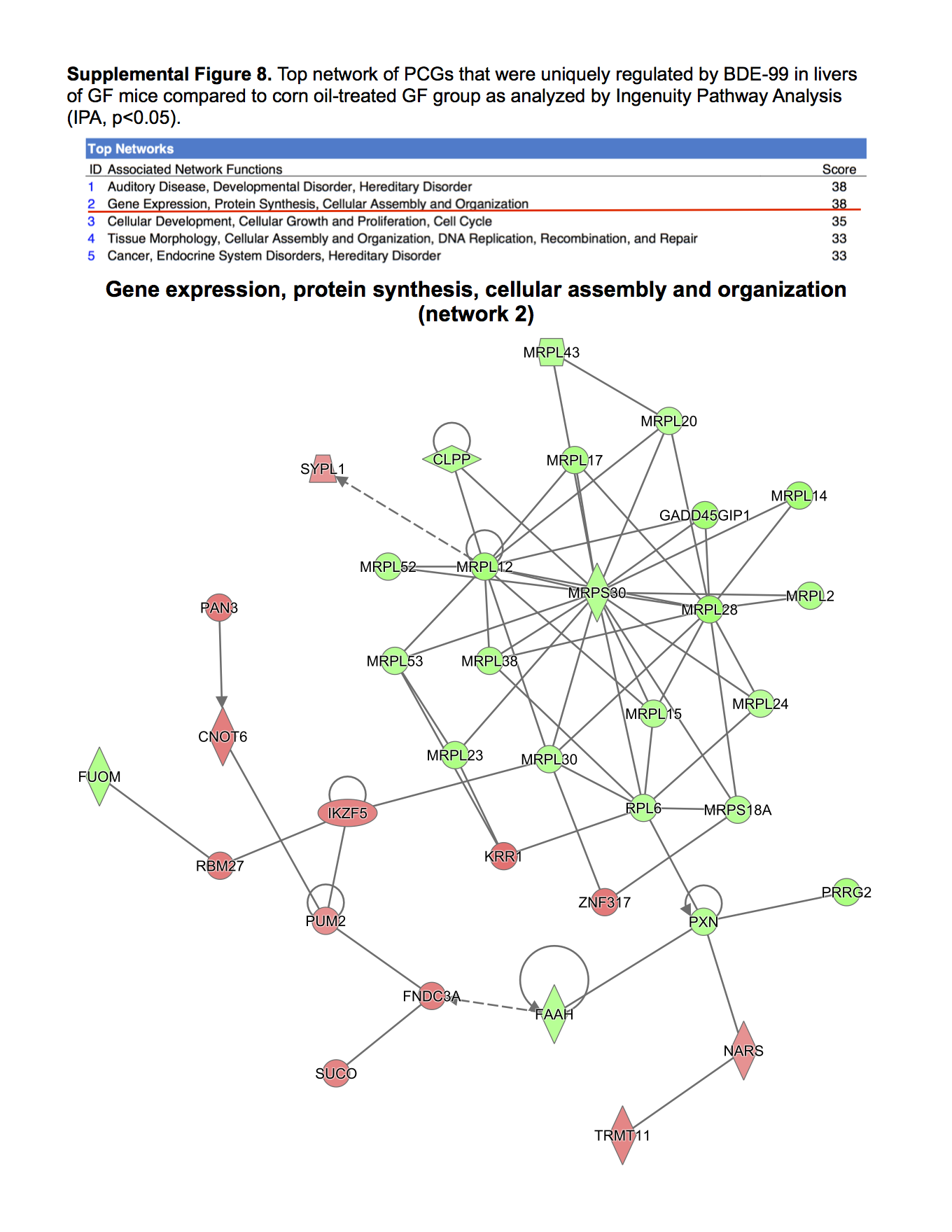

Supplement: S8 Fig — (TIFF) [file pone.0201387.s008.tiff]

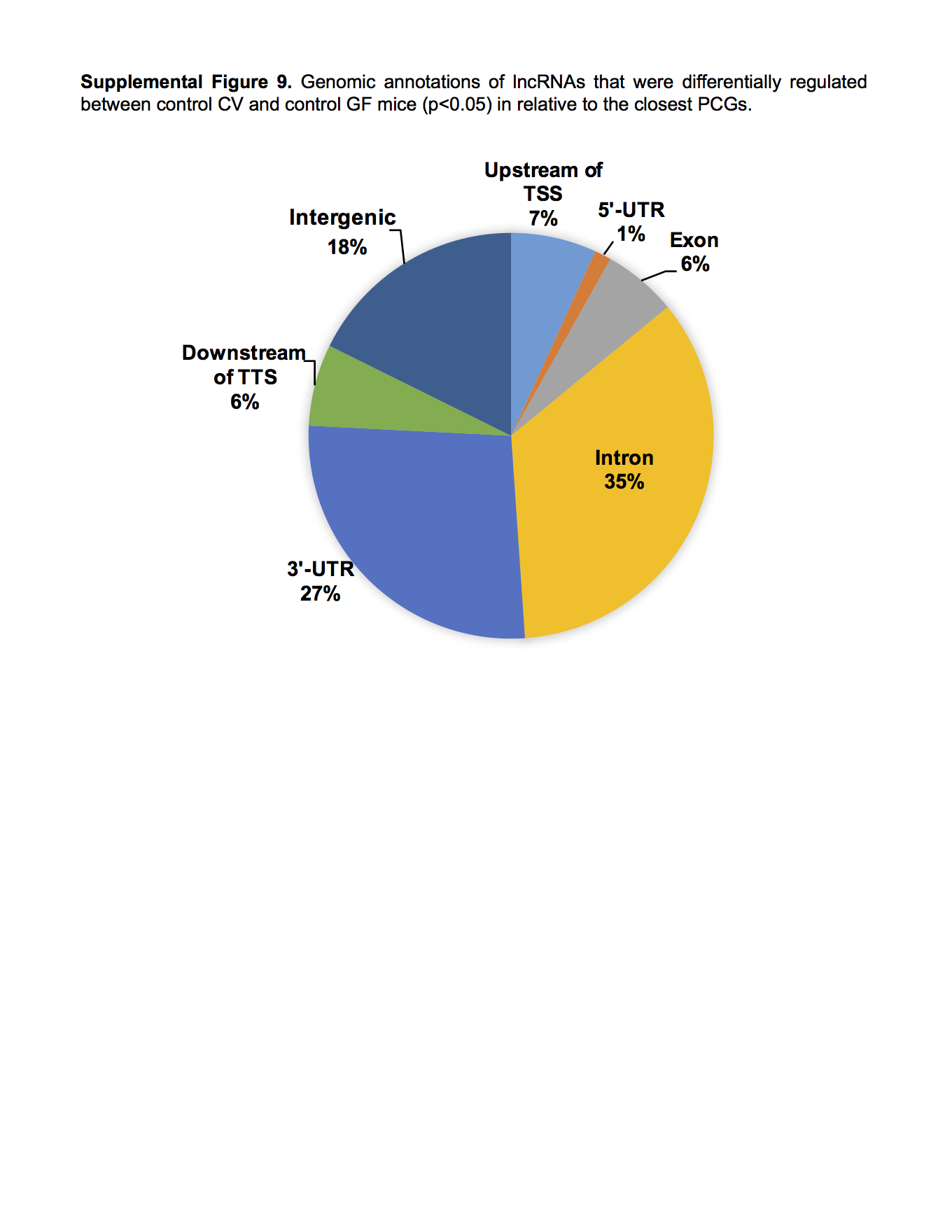

Supplement: S9 Fig — (TIFF) [file pone.0201387.s009.tiff]

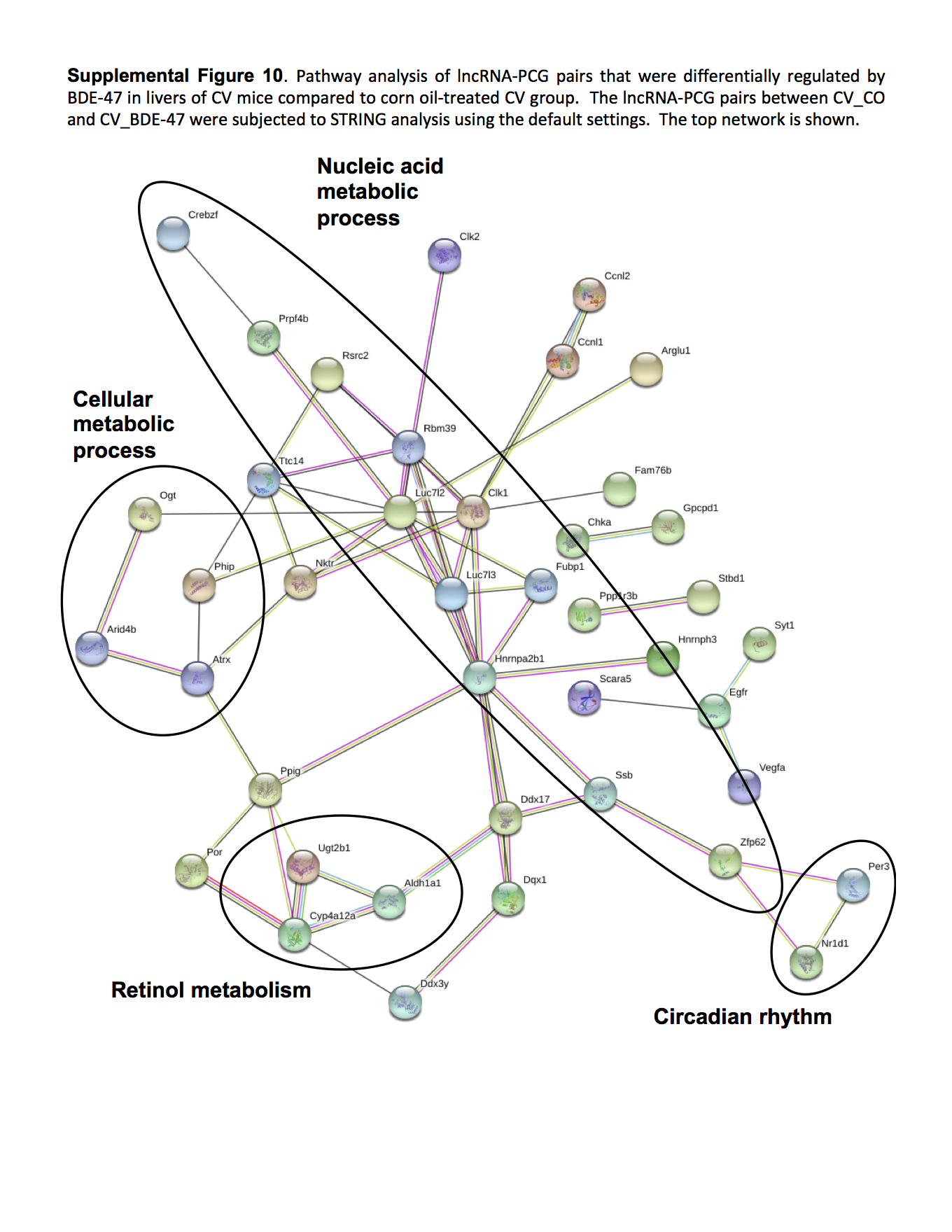

Supplement: S10 Fig — The lncRNA-PCG pairs between CV_CO and CV_BDE-47 were subjected to STRING analysis using the default settings. The top network is shown. (TIFF) [file pone.0201387.s010.tiff]

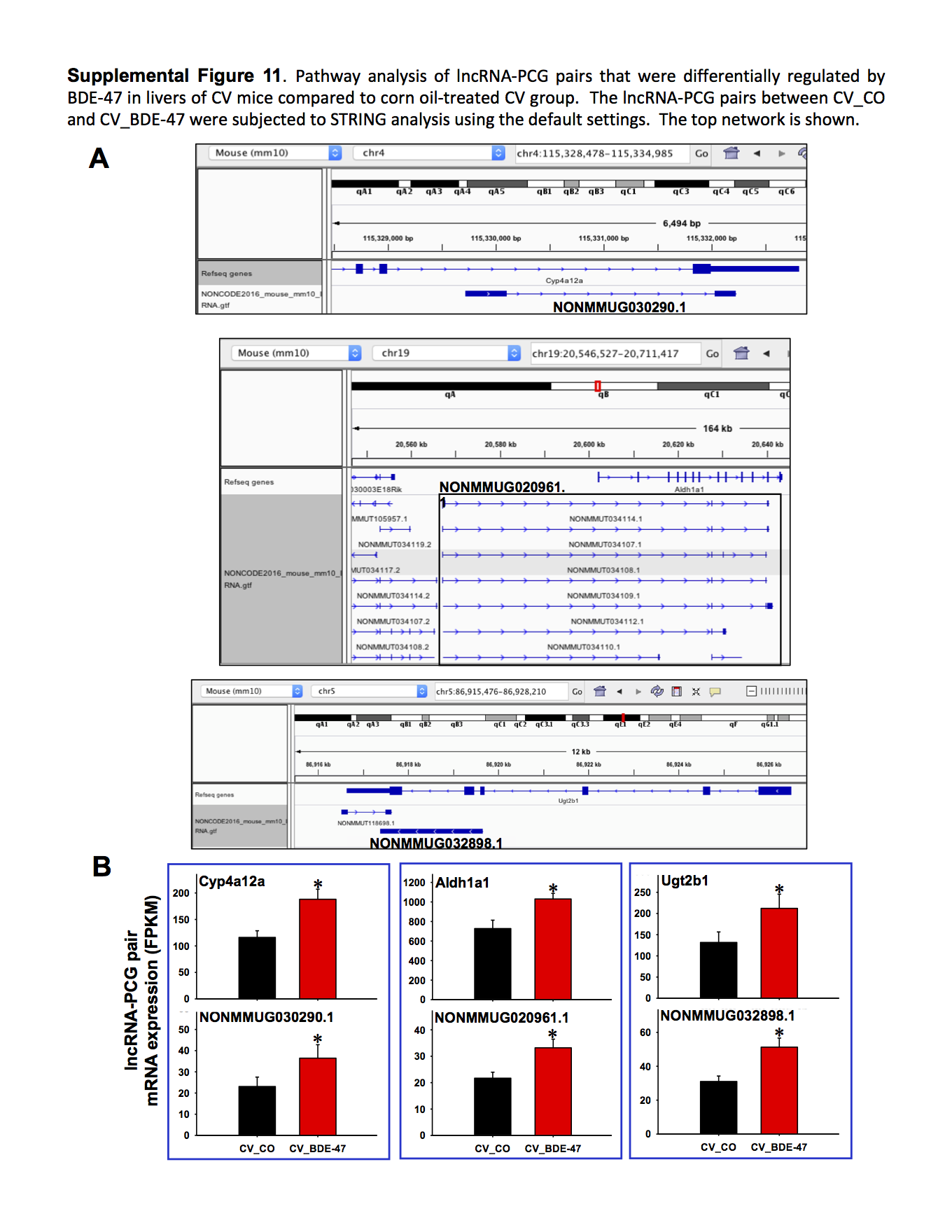

Supplement: S11 Fig — Genomic location (A) and gene expression (B) of lncRNA-PCG pairs that were differentially regulated by BDE-47 in livers of CV mice compared to corn oil-treated CV mice. Drug-metabolizing enzymes Cyp4a12a and aldehyde dehydrogenase (Aldh) 1a1 (phase I), as well as UDP-glucuronosyltransferase (Ugt) 2b1 (phase II) are shown. Expression of lncRNAs and paired PCGs were plotted using mean FPKM ± S.E. Asterisks (*) indicate statistically significant differences as compared to vehicle-treated groups of the same enterotypes of mice (p < 0.05). (TIFF) [file pone.0201387.s011.tiff]

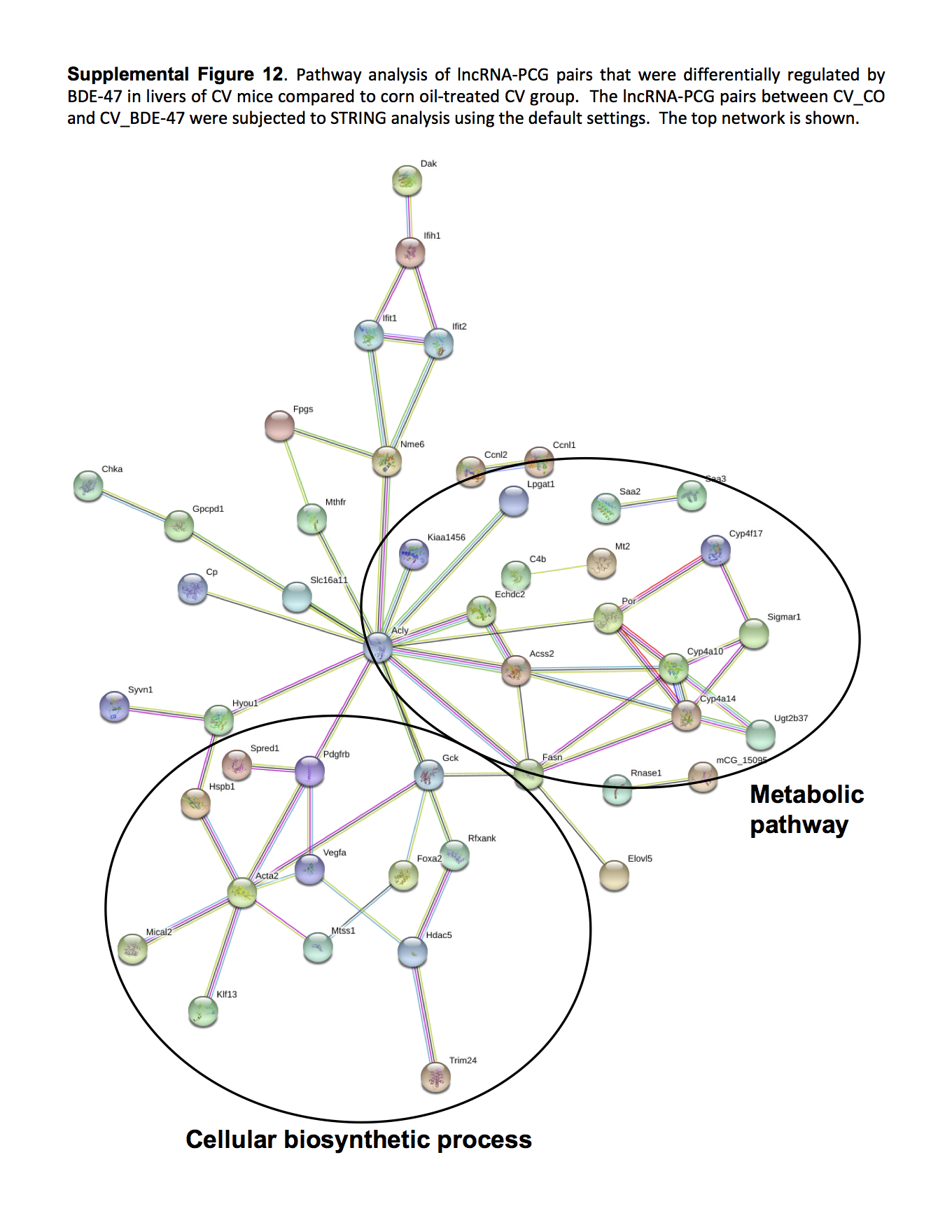

Supplement: S12 Fig — The lncRNA-PCG pairs between CV_CO and CV_BDE-99 were subjected to STRING analysis using the default settings. The top network is shown. (TIFF) [file pone.0201387.s012.tiff]

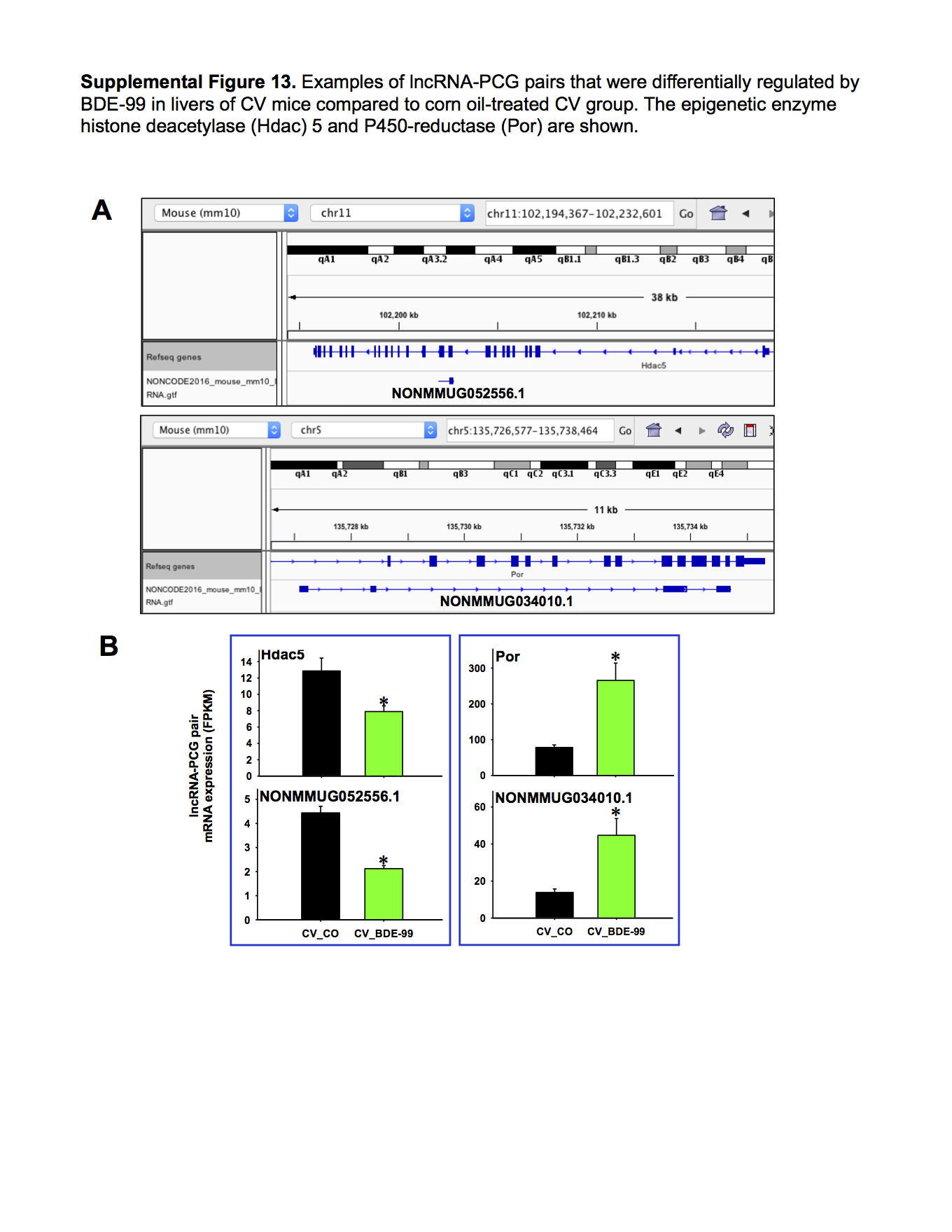

Supplement: S13 Fig — Genomic location (A) and gene expression (B) of lncRNA-PCG pairs lncRNA-PCG pairs that were differentially regulated by BDE-99 in livers of CV mice compared to corn oil-treated CV group. The epigenetic enzyme histone deacetylase (Hdac) 5 and P450-reductase (Por) are shown. (TIFF) [file pone.0201387.s013.tiff]

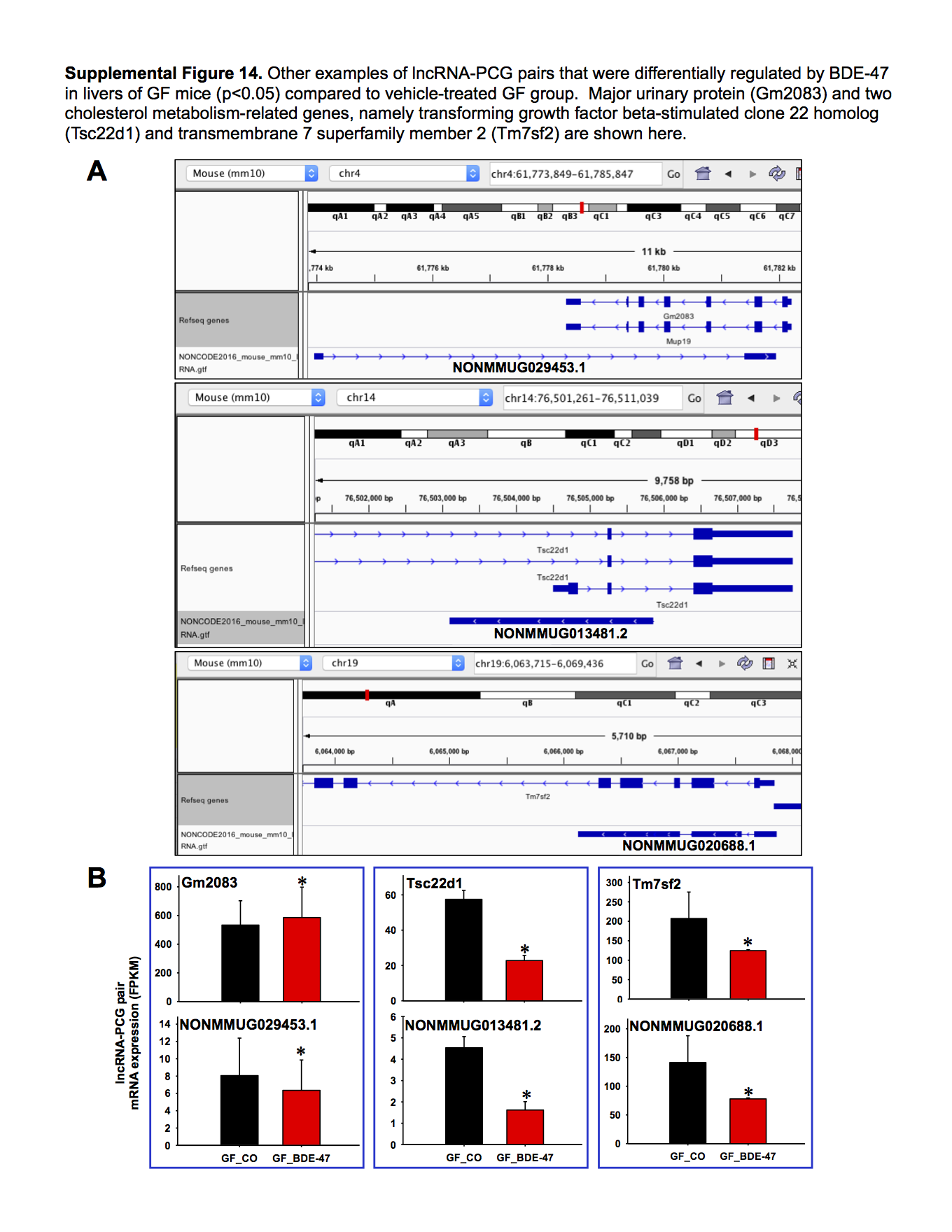

Supplement: S14 Fig — Genomic location (A) and gene expression (B) of lncRNA-PCG pairs lncRNA-PCG pairs differentially regulated by BDE-47 in livers of GF mice (p<0.05) compared to vehicle-treated GF group. Major urinary protein (Gm2083) and two cholesterol metabolism-related genes, namely transforming growth factor beta-stimulated clone 22 homolog (Tsc22d1) and transmembrane 7 superfamily member 2 (Tm7sf2, also known as delta (14)-sterol reductase) are shown here. (TIFF) [file pone.0201387.s014.tiff]

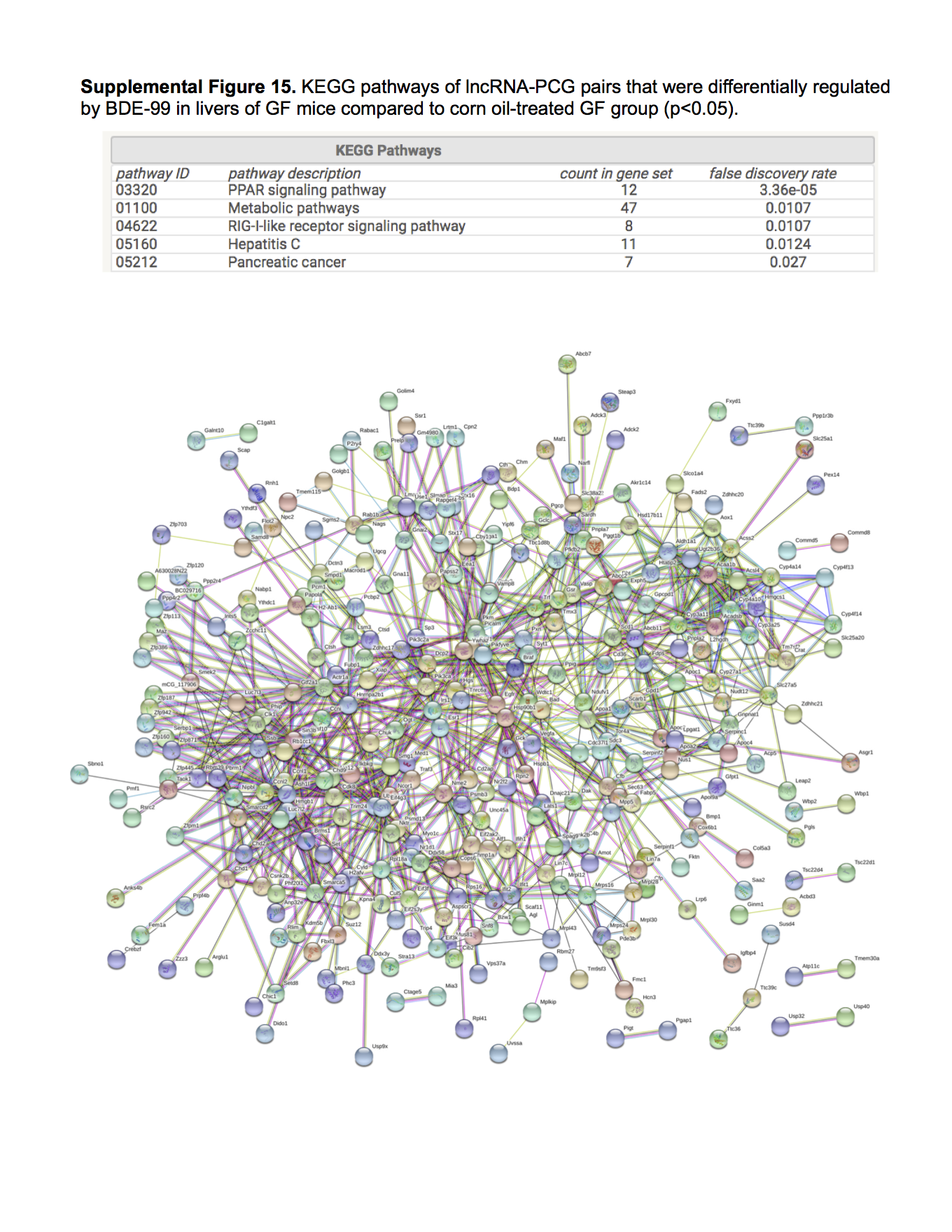

Supplement: S15 Fig — (TIFF) [file pone.0201387.s015.tiff]

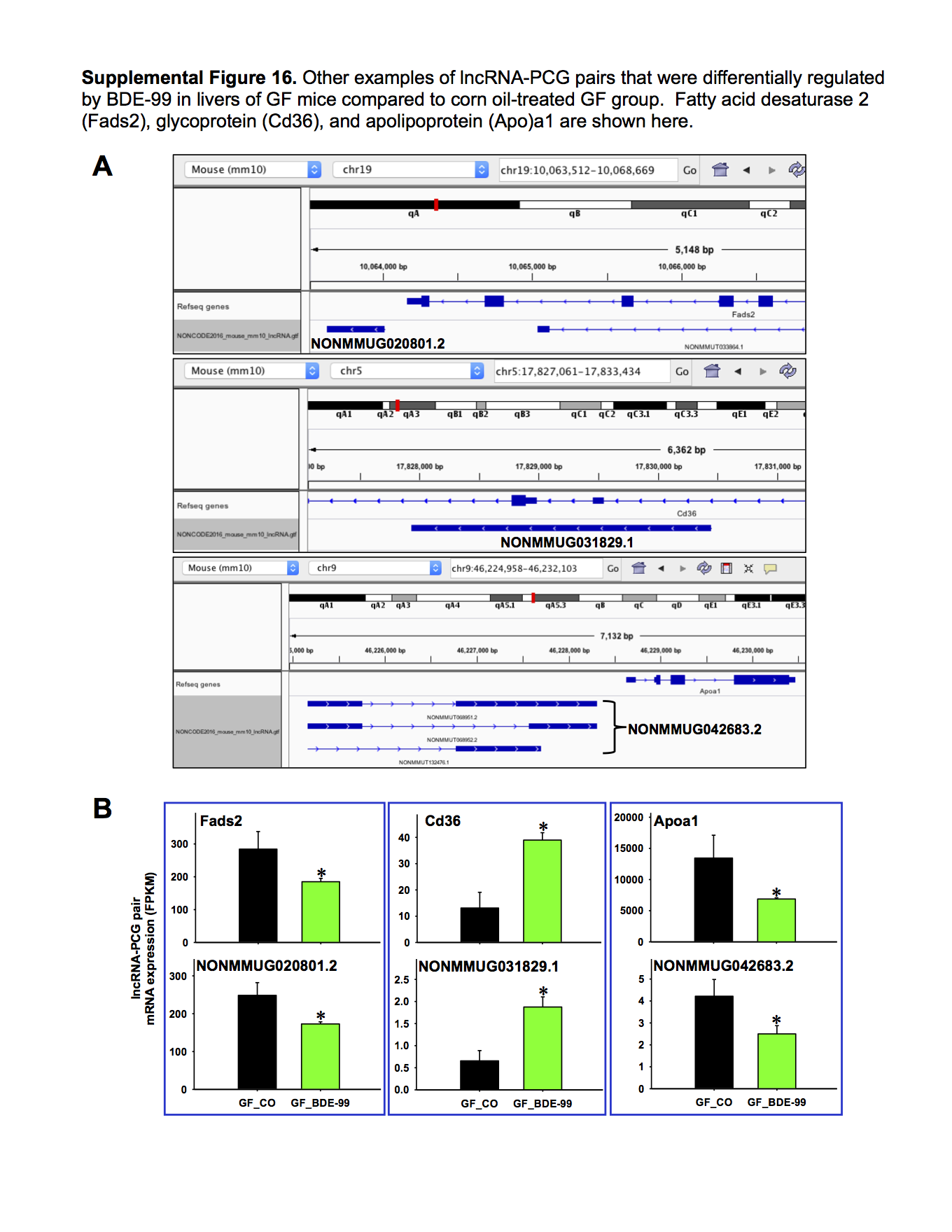

Supplement: S16 Fig — Genomic location (A) and gene expression (B) of lncRNA-PCG pairs lncRNA-PCG pairs differentially regulated by BDE-99 in livers of GF mice compared to corn oil-treated GF group. Fatty acid desaturase 2 (Fads2), glycoprotein (Cd36), and apolipoprotein (Apo)a1 are shown here. (TIFF) [file pone.0201387.s016.tiff]

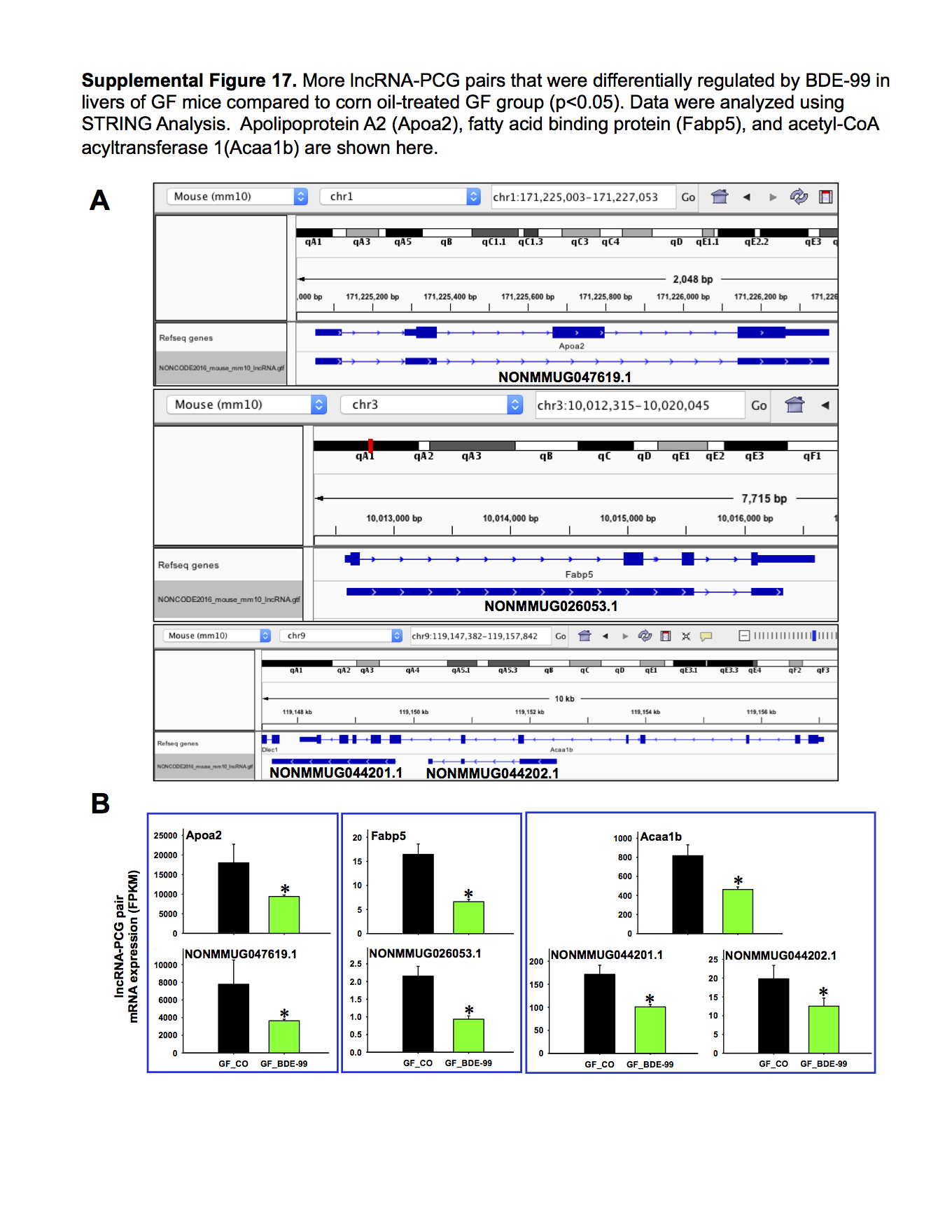

Supplement: S17 Fig — Genomic location (A) and gene expression (B) of lncRNA-PCG pairs lncRNA-PCG pairs differentially regulated by BDE-99 in livers of GF mice compared to corn oil-treated GF group (p<0.05). Data were analyzed using STRING Analysis. Apolipoprotein A2 (Apoa2), fatty acid binding protein (Fabp5), and acetyl- CoA acyltransferase 1(Acaa1b) are shown here. (TIFF) [file pone.0201387.s017.tiff]

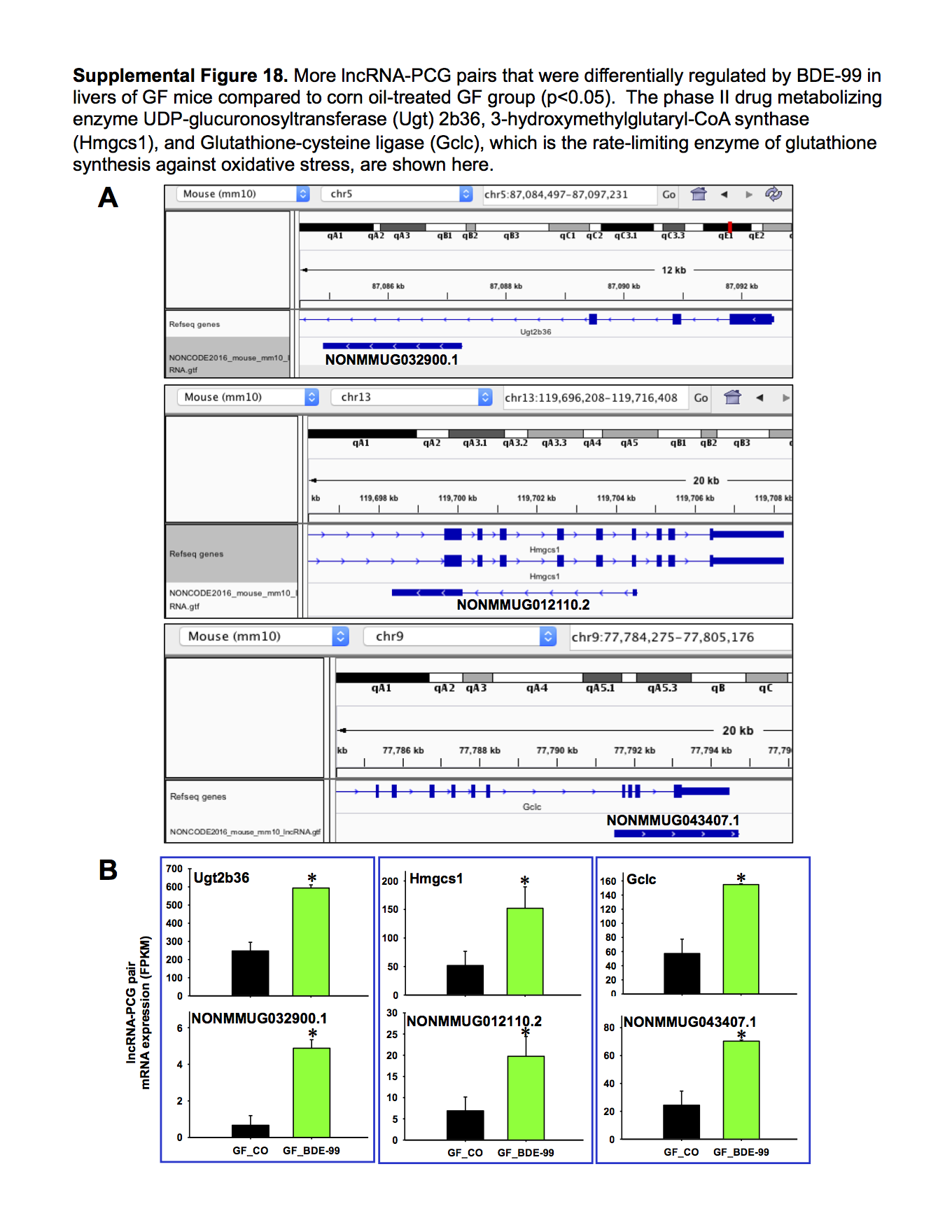

Supplement: S18 Fig — Genomic location (A) and gene expression (B) of lncRNA-PCG pairs lncRNA-PCG pairs differentially regulated by BDE-99 in livers of GF mice compared to corn oil-treated GF group (p<0.05). The phase II drug metabolizing enzyme UDP-glucuronosyltransferase (Ugt) 2b36, 3-hydroxymethylglutaryl-CoA synthase (Hmgcs1), and Glutathione-cysteine ligase (Gclc), which is the rate-limiting enzyme of glutathione synthesis against oxidative stress, are shown here. (TIFF) [file pone.0201387.s018.tiff]

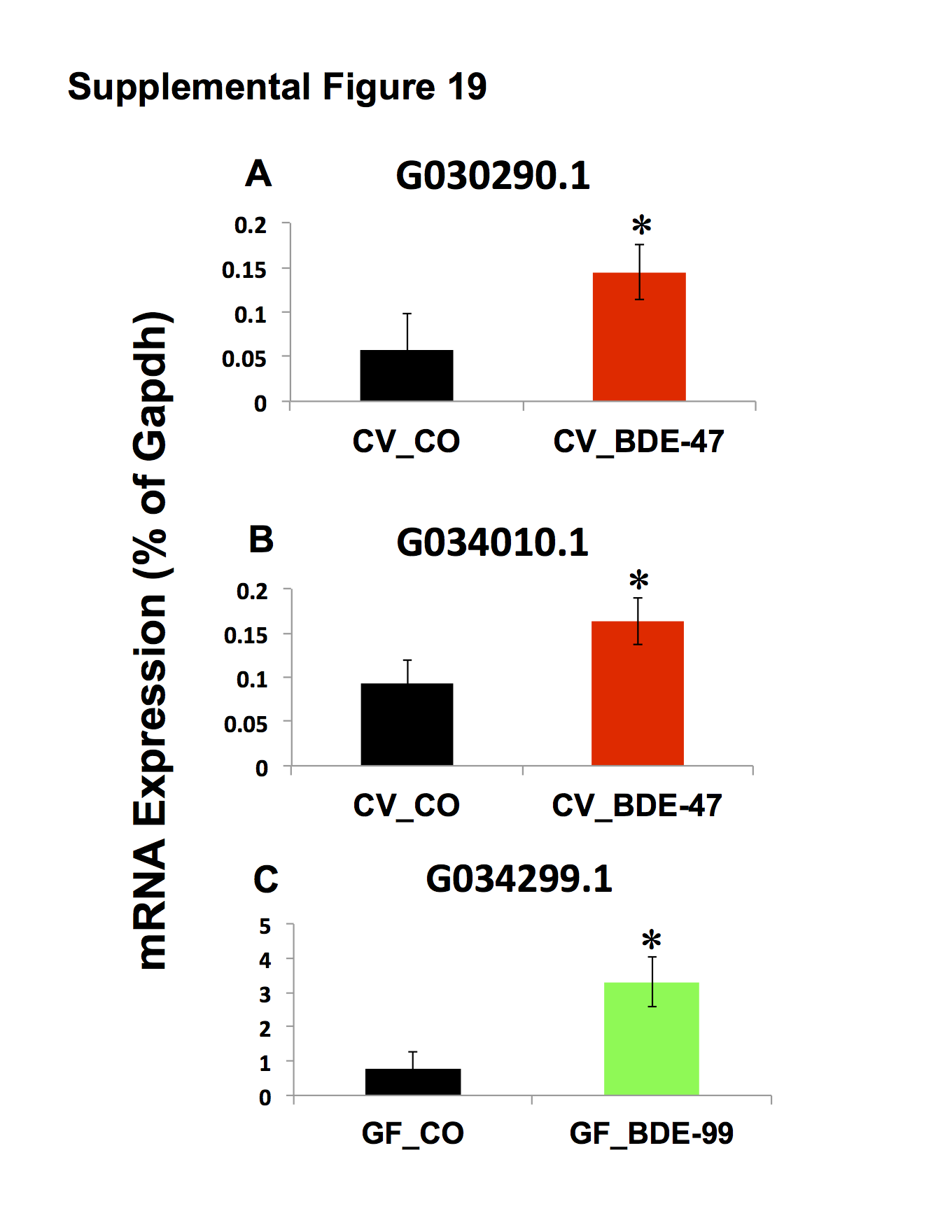

Supplement: S19 Fig — A. Expression of the lncRNA G030290.1 (paired with the fatty acid metabolizing enzyme Cyp4a12a, S11 Fig). B. Expression of the lncRNA G034010.1 (paired with the P450 reductase Por, Fig 5). C. Expression of the lncRNA G034299.1 (paired with Cyp3a25, Fig 6B). Data are expressed as % of the house-keeping gene Gapdh. Asterisks represent statistically significant differences as compared to the vehicle-treated group (p<0.05). (TIFF) [file pone.0201387.s019.tiff]
