# Supplementary material for: Regulation of protein-coding gene and long noncoding RNA pairs in liver of conventional and germ-free mice following oral PBDE exposure
Source: PLoS One. 2018 Aug 1;13(8):e0201387. doi: 10.1371/journal.pone.0201387 (PMC6070246; doi:10.1371/journal.pone.0201387)
Supplement: S1 Table — (PDF) [file pone.0201387.s020.pdf]

Supplemental Materials

Title: Gut microbiome modifies the PBDE-mediated coordinate regulation of protein-coding genes and long non-coding RNAs in livers of conventional and germ-free mice

Cindy Yanfei Li and Julia Yue Cui*

Department of Environmental and Occupational Health Sciences, University of Washington, Seattle, WA 98105

Running title: Regulation of hepatic transcriptome by gut microbiome and PBDEs

* Corresponding author
Email: juliacui@uw.edu (J.Y.C)

II. Supplemental Tables

Supplemental Table 1. Mapping statistics of RNA-Seq.

Sample	Total reads	reads mapped	% mapped
CV_CO1	127166234	114920125	90.37%
CV_CO2	72155698	52298449	72.48%
CV_CO3	65480436	55736947	85.12%
CV_BDE47_1	115222113	91751368	79.63%
CV_BDE47_2	65093299	56155989	86.27%
CV_BDE47_3	48123870	41131471	85.47%
CV_BDE99_1	105371498	97936933	92.94%
CV_BDE99_2	46057295	42014130	91.22%
GF_CO1	92574095	86584551	93.53%
GF_CO2	84704938	53528596	63.19%
GF_CO3	47418827	43114075	90.92%
GF_BDE47_1	82417373	76999864	93.43%
GF_BDE47_2	38614284	35522885	91.99%
GF_BDE47_3	49942704	38686913	77.46%
GF_BDE99_1	48804600	44545654	91.27%
GF_BDE99_2	50005307	46728929	93.45%
GF_BDE99_3	44559087	41316843	92.72%
