# Supplementary material for: Regulation of protein-coding gene and long noncoding RNA pairs in liver of conventional and germ-free mice following oral PBDE exposure
Source: PLoS One. 2018 Aug 1;13(8):e0201387. doi: 10.1371/journal.pone.0201387 (PMC6070246; doi:10.1371/journal.pone.0201387)
Supplement: S2 Table — The network in red color was shown in Supplemental Figures. (PDF) [file pone.0201387.s021.pdf]

Supplemental Table 2. Top 15 networks for PCGs that were differentially regulated by PBDEs in livers of CV and GF mice compared to corn oil-treated control group of the same enterotype of mice. The network in red color was shown in Supplemental Figures.

CV_CO vs CV_BDE-47:

	**Top Networks**	**Focus Molecules**
1	RNA Post-Transcriptional Modification, Developmental Disorder, Hereditary Disorder	34
2	Cellular Development, Cellular Growth and Proliferation, Cell Cycle	32
3	Cancer, Gastrointestinal Disease, Organismal Injury and Abnormalities	30
4	Behavior, Nervous System Development and Function, Antimicrobial Response	27
5	Cancer, Organismal Injury and Abnormalities, Renal and Urological Disease	27
6	Cell Cycle, Gene Expression, Cellular Growth and Proliferation	26
7	Cancer, Cellular Movement, Organismal Injury and Abnormalities	26
8	Lipid Metabolism, Small Molecule Biochemistry, Endocrine System Development and Function	25
9	Dermatological Diseases and Conditions, Organismal Injury and Abnormalities, DNA Replication, Recombination, and Repair	24
10	Carbohydrate Metabolism, Molecular Transport, Cell-To-Cell Signaling and Interaction	24
11	Immunological Disease, Antimicrobial Response, Inflammatory Response	24
12	RNA Damage and Repair, Respiratory System Development and Function, Cancer	23
13	Carbohydrate Metabolism, Cell Morphology, Cellular Assembly and Organization	23
14	Lipid Metabolism, Small Molecule Biochemistry, Drug Metabolism	22
15	Cell Cycle, Cellular Assembly and Organization, DNA Replication, Recombination, and Repair	21

CV_CO vs CV_BDE-99:

	**Top Networks**	**Focus Molecules**
1	Lipid Metabolism, Small Molecule Biochemistry, Molecular Transport	28
2	Behavior, Nervous System Development and Function, Gene Expression	26
3	Drug Metabolism, Protein Synthesis, Glutathione Depletion In Liver	26
4	RNA Post-Transcriptional Modification, Cell Cycle, Connective Tissue Development and Function	26
5	Endocrine System Disorders, Gastrointestinal Disease, Immunological Disease	26
6	Lipid Metabolism, Small Molecule Biochemistry, Vitamin and Mineral Metabolism	25

7	Gene Expression, Lipid Metabolism, Molecular Transport	24
8	Carbohydrate Metabolism, Cellular Development, Cellular Growth and Proliferation	24
9	Cancer, Connective Tissue Disorders, Endocrine System Disorders	23
10	Endocrine System Disorders, Gastrointestinal Disease, Immunological Disease	22
11	Cardiovascular System Development and Function, Cardiovascular Disease, Cancer	22
12	Cancer, Connective Tissue Disorders, Organismal Injury and Abnormalities	21
13	Lipid Metabolism, Small Molecule Biochemistry, Vitamin and Mineral Metabolism	20
14	Immunological Disease, Endocrine System Disorders, Gastrointestinal Disease	20
15	Cellular Compromise, Cellular Development, Embryonic Development	20

GF_CO vs GF_BDE-47:

	**Top Networks**	**Focus Molecules**
1	Drug Metabolism, Glutathione Depletion In Liver, Lipid Metabolism	21
2	Lipid Metabolism, Liver Cholestasis, Molecular Transport	21
3	Drug Metabolism, Small Molecule Biochemistry, Cell-To-Cell Signaling and Interaction	19
4	Endocrine System Development and Function, Small Molecule Biochemistry, Drug Metabolism	15
5	Carbohydrate Metabolism, Digestive System Development and Function, Cardiovascular Disease	14
6	Cell-To-Cell Signaling and Interaction, Cell-mediated Immune Response, Cellular Movement	14
7	Organ Development, Reproductive System Development and Function, Developmental Disorder	13
8	Cellular Growth and Proliferation, Lymphoid Tissue Structure and Development, Cellular Development	12
9	Cell-To-Cell Signaling and Interaction, Molecular Transport, Small Molecule Biochemistry	11
10	Cell-To-Cell Signaling and Interaction, Nervous System Development and Function, Cellular Growth and Proliferation	10
11	Embryonic Development, Organismal Development, Tissue Development	10
12	Cell Cycle, Cellular Assembly and Organization, Cancer	1

GF_CO vs GF_BDE-99:

	**Top Networks**	**Focus Molecules**
1	Cancer, Organismal Injury and Abnormalities, Hereditary Disorder	35
2	Developmental Disorder, Hereditary Disorder, Metabolic Disease	35
